# Supplementary material for: Basal State Calibration of a Chemical Reaction Network Model for Autophagy
Source: Int J Mol Sci. 2024 Oct 21;25(20):11316. doi: 10.3390/ijms252011316 (PMC11508741; doi:10.3390/ijms252011316)
Supplement: Supplementary file 1 [file ijms-25-11316-s001.zip › ijms-3228738-supplementary.pdf]

1 Liu et al. model's original reactions

The calculations were carried out in $\frac{mol}{cm^3}$, thus the rate coefficients values for the first and second order reactions are taken in $\frac{1}{s}$ and $\frac{cm^3}{mol*s}$ units, respectively.

$$\mathrm{AMPK} + \mathrm{nS} \xrightarrow{k0} \mathrm{AMPKa} + \mathrm{nS}$$
$$\mathrm{AMPKa} \xrightarrow{k1} \mathrm{AMPK}$$
$$\mathrm{AMPKa} + \mathrm{mTORa} \xrightarrow{k2} \mathrm{AMPKa} + \mathrm{mTOR}$$
$$\mathrm{AMPKa} + \mathrm{mTORa\_ULKa} \xrightarrow{k3} \mathrm{AMPKa} + \mathrm{mTOR} + \mathrm{ULKa}$$
$$\mathrm{mTORa} \xrightarrow{k4} \mathrm{mTOR}$$
$$\mathrm{mTORa} + \mathrm{AMPKa} \xrightarrow{k5} \mathrm{mTORa} + \mathrm{AMPK}$$
$$\mathrm{ULK} + \mathrm{mTORa} \underset{k7}{\overset{k6}{\rightleftharpoons}} \mathrm{mTORa\_ULK}$$
$$\mathrm{ULKa} + \mathrm{mTORa} \underset{k9}{\overset{k8}{\rightleftharpoons}} \mathrm{mTORa\_ULK}$$
$$\mathrm{AMPKa} + \mathrm{ULK} \xrightarrow{k10} \mathrm{AMPKa} + \mathrm{ULKa}$$
$$\mathrm{ULKa} \xrightarrow{k11} \mathrm{ULKa} + \mathrm{phag}$$
$$\mathrm{AMPKa} + \mathrm{TSC} \xrightarrow{k12} \mathrm{AMPKa} + \mathrm{TSCa}$$
$$\mathrm{RHEBa} + \mathrm{TSCa} \xrightarrow{k13} \mathrm{RHEB} + \mathrm{TSCa}$$
$$\mathrm{RHEBa} + \mathrm{mTOR} \xrightarrow{k14} \mathrm{RHEBa} + \mathrm{mTORa}$$
$$\mathrm{RHEB} \xrightarrow{k15} \mathrm{RHEBa}$$
$$\mathrm{ULKa} \xrightarrow{k16} \mathrm{ULK}$$
$$\mathrm{AMPKa} + \mathrm{ULKa} \xrightarrow{k17} \mathrm{AMPK} + \mathrm{ULKa}$$
$$\mathrm{phag} + \mathrm{BEC1} \xrightarrow{k18} \mathrm{preAUT} + \mathrm{BEC1}$$
$$\mathrm{phag} + \mathrm{BEC1\_UVG} \xrightarrow{k19} \mathrm{preAUT} + \mathrm{BEC1\_UVG}$$
$$\mathrm{BEC1} + \mathrm{UVG} \underset{k21}{\overset{k20}{\rightleftharpoons}} \mathrm{BEC1\_UVG}$$
$$\varnothing \xrightarrow{k22} \mathrm{BEC1}$$
$$\mathrm{BEC1c} \xrightarrow{k23} \mathrm{cytc} + \mathrm{BEC1c}$$
$$\mathrm{BEC1c} \xrightarrow{k24} \varnothing$$
$$\mathrm{phag} \xrightarrow{k25} \varnothing$$
$$\mathrm{ATG5} + \mathrm{phag} \xrightarrow{k26} \mathrm{ATG5} + \mathrm{AUT}$$
$$\mathrm{AUT} \xrightarrow{k27} \varnothing$$
$$\mathrm{AUT} + \mathrm{nS} \xrightarrow{k28} \mathrm{AUT}$$
$$\mathrm{AUT} + \mathrm{eS} \xrightarrow{k29} \mathrm{AUT}$$

$$\varnothing \xrightarrow{k30} \text{phag}$$

$$\text{eS} + \text{PERK} \xrightarrow{k31} \text{PERKa}$$

$$\text{ATF4a} \xrightarrow{k32} \text{phag}$$

$$\text{PERKa} + \text{ATF4} \xrightarrow{k33} \text{PERK} + \text{ATF4a}$$

$$\text{preAUT} \xrightarrow{k34} \varnothing$$

$$\text{dS} + \text{DAPK} \xrightarrow{k35} \text{dS} + \text{DAPKa}$$

$$\text{DAPKa} \xrightarrow{k36} \text{DAPK}$$

$$\text{EPACa} + \text{PLCe} \xrightarrow{k37} \text{EPACa} + \text{PLCea}$$

$$\text{PLCea} \xrightarrow{k38} \text{PLCe}$$

$$\text{PLCea} + \text{PIP2} \xrightarrow{k39} \text{PLCea} + \text{IP3}$$

$$\text{Ca2er} + \text{IP3R\_IP3} \xrightarrow{k40} \text{Ca2ic} + \text{IP3R\_IP3}$$

$$\text{Ca2ic} + \text{SERCA} \xrightarrow{k41} \text{Ca2er} + \text{SERCA}$$

$$\text{Ca2ic} + \text{Bax} \xrightarrow{k42} \text{Ca2ic} + \text{Baxa}$$

$$\text{IP3R} + \text{IP3} \xrightleftharpoons[k44]{k43} \text{IP3R\_IP3}$$

$$\text{IP3} \xrightarrow{k45} \text{IP2}$$

$$\text{IP2} \xrightarrow{k46} \text{IP}$$

$$\text{IP} \xrightarrow{k47} \text{Ins}$$

$$\text{Ins} \xrightarrow{k48} \text{PIP2}$$

$$\text{IP3R\_IP3} + \text{phag} \xrightarrow{k49} \text{IP3R\_IP3}$$

$$\text{Ca2ic} + \text{CaMKKb} \xrightarrow{k50} \text{Ca2ic} + \text{CaMKKba}$$

$$\text{CaMKKba} \xrightarrow{k51} \text{Ca2ic} + \text{CaMKKb}$$

$$\text{AMPK} + \text{CaMKKba} \xrightarrow{k52} \text{AMPKa} + \text{CaMKKba}$$

$$\text{IP3R} + \text{BCL2} \xrightleftharpoons[k54]{k53} \text{IP3R\_BCL2}$$

$$\text{BEC1} + \text{BCL2} \xrightleftharpoons[k56]{k55} \text{BEC1\_BCL2}$$

$$\text{BCL2p} \xrightarrow{k57} \text{BCL2}$$

$$\text{Ca2ic} + \text{Calpain} \xrightarrow{k58} \text{Ca2ic} + \text{Calpaina}$$

$$\text{Calpaina} \xrightarrow{k59} \text{Calpain}$$

$$\text{Calpaina} + \text{Ga} \xrightarrow{k60} \text{Gaa} + \text{Calpaina}$$

$$\text{Calpaina} + \text{ATG5} \xrightarrow{k61} \text{Calpaina} + \text{ATG5t}$$

$$\text{ATG5t} + \text{BCL2} \xrightleftharpoons[k63]{k62} \text{ATG5\_BCL2}$$

$$\text{Calpaina} + \text{Bid} \xrightarrow{k64} \text{Calpaina} + \text{tBid}$$

$$\text{Calpaina} + \text{BEC1} \xrightarrow{k65} \text{Calpaina}$$

$$\text{AC} + \text{Gaa} \underset{k67}{\overset{k66}{\rightleftharpoons}} \text{AC\_Gaa}$$

$$\text{AC\_Gaa} \xrightarrow{k68} \text{AC\_Gaa} + \text{cAMP}$$

$$\text{cAMP} \xrightarrow{k69} \varnothing$$

$$\text{cAMP} + \text{EPAC} \xrightarrow{k70} \text{cAMP} + \text{EPACa}$$

$$\text{EPACa} \xrightarrow{k71} \text{EPAC}$$

$$\text{Ga} \xrightarrow{k72} \text{G}$$

$$\text{AC} + \text{Ga} \underset{k74}{\overset{k73}{\rightleftharpoons}} \text{AC\_Ga}$$

$$\text{Gabg} + \text{GPCRa} \xrightarrow{k75} \text{Ga} + \text{Gbg} + \text{GPCRa}$$

$$\text{Ga} + \text{Gbg} \xrightarrow{k76} \text{Gabg}$$

$$\text{BCL2} + \text{Bax} \underset{k78}{\overset{k77}{\rightleftharpoons}} \text{BCL2\_Bax}$$

$$\text{Bax} + \text{p53m} \xrightarrow{k79} \text{Baxa} + \text{p53m}$$

$$\text{Baxa} \xrightarrow{k80} \text{Bax}$$

$$\text{Bax} \xrightarrow{k81} \varnothing$$

$$\text{UVG} + \text{Bax} \underset{k83}{\overset{k82}{\rightleftharpoons}} \text{UVG\_Bax}$$

$$\text{cytc} + \text{caspase} \xrightarrow{k84} \text{cytc} + \text{caspasea}$$

$$\text{Bid} + \text{caspasea} \xrightarrow{k85} \text{tBid} + \text{caspasea}$$

$$\text{tBid} \xrightarrow{k86} \varnothing$$

$$\varnothing \xrightarrow{k87} \text{Bid}$$

$$\text{JNKa} + \text{caspase} \xrightarrow{k88} \text{JNKa} + \text{caspasea}$$

$$\text{dS} \xrightarrow{k89} \text{dS} + \text{p53c}$$

$$\text{p53c} \xrightarrow{k90} \varnothing$$

$$\text{p53c} \underset{k92}{\overset{k91}{\rightleftharpoons}} \text{p53a}$$

$$\text{p53a} \xrightarrow{k93} \text{p53a} + \text{Mdm2}$$

$$\text{p53c} + \text{Mdm2} \xrightarrow{k94} \text{p53m} + \text{Mdm2}$$

$$\text{p53a} \xrightarrow{k95} \text{p53a} + \text{Bax}$$

$$\text{p53m} + \text{BCL2} \underset{k97}{\overset{k96}{\rightleftharpoons}} \text{p53\_BCL2}$$

$$\text{Mdm2} \xrightarrow{k98} \varnothing$$

$$\text{p53a} \xrightarrow{k99} \text{p53a} + \text{DRAM}$$

$$\mathrm{DRAM} \xrightarrow{k100} \varnothing$$

$$\mathrm{DRAM} + \mathrm{preAUT} \xrightarrow{k101} \mathrm{DRAM} + \mathrm{AUT}$$

$$\mathrm{p53c} + \mathrm{AMPKa} \xrightarrow{k102} \mathrm{p53c} + \mathrm{AMPK}$$

$$\mathrm{p53a} \xrightarrow{k103} \mathrm{p53a} + \mathrm{PUMA}$$

$$\mathrm{PUMA} + \mathrm{BCL2} \underset{k105}{\overset{k104}{\rightleftharpoons}} \mathrm{PUMA\_BCL2}$$

$$\mathrm{PUMA} \xrightarrow{k106} \varnothing$$

$$\mathrm{Baxa} \xrightarrow{k107} \mathrm{cytc} + \mathrm{Baxa}$$

$$\mathrm{cytc} \xrightarrow{k108} \varnothing$$

$$\mathrm{mTORa} + \mathrm{TFEBi} \xrightarrow{k109} \mathrm{mTORa} + \mathrm{TFEB}$$

$$\mathrm{phag} + \mathrm{TFEB} \xrightarrow{k110} \mathrm{AUT} + \mathrm{TFEB}$$

$$\mathrm{TFEB} \xrightarrow{k111} \mathrm{TFEBi}$$

$$\mathrm{Ins} + \mathrm{PI3K} \xrightarrow{k112} \mathrm{Ins} + \mathrm{PI3Ka}$$

$$\mathrm{PI3Ka} \xrightarrow{k113} \mathrm{PI3K}$$

$$\mathrm{AKT} + \mathrm{PI3Ka} \xrightarrow{k114} \mathrm{AKTa} + \mathrm{PI3K}$$

$$\mathrm{AKTa} \xrightarrow{k115} \mathrm{AKT}$$

$$\mathrm{AKTa} + \mathrm{TSCa} \xrightarrow{k116} \mathrm{AKTa} + \mathrm{TSC}$$

$$\mathrm{AKTa} + \mathrm{mTORa\_ULKa} \xrightarrow{k117} \mathrm{AKTa} + \mathrm{mTOR} + \mathrm{ULKa}$$

$$\mathrm{JNKa} + \mathrm{BCL2} \xrightarrow{k118} \mathrm{JNKa} + \mathrm{BCL2p}$$

$$\mathrm{JNKa} + \mathrm{BEC1\_BCL2} \xrightarrow{k119} \mathrm{JNKa} + \mathrm{BEC1} + \mathrm{BCL2p}$$

$$\mathrm{DAPKa} + \mathrm{BEC1\_BCL2} \xrightarrow{k120} \mathrm{DAPKa} + \mathrm{BEC1} + \mathrm{BCL2}$$

$$\mathrm{eS} + \mathrm{JNK} \xrightarrow{k121} \mathrm{eS} + \mathrm{JNKa}$$

$$\mathrm{JNKa} \xrightarrow{k122} \mathrm{JNK}$$

$$\mathrm{nS} + \mathrm{MAPK15} \xrightarrow{k123} \mathrm{nS} + \mathrm{MAPK15a}$$

$$\mathrm{MAPK15a} \xrightarrow{k124} \mathrm{MAPK15}$$

$$\mathrm{PKA} + \mathrm{MAPK15a} \xrightarrow{k125} \mathrm{PKAa} + \mathrm{MAPK15a}$$

$$\mathrm{PKAa} \xrightarrow{k126} \mathrm{PKA}$$

$$\mathrm{PKAa} + \mathrm{phag} \xrightarrow{k127} \mathrm{PKAa} + \mathrm{preAUT}$$

$$\mathrm{PKAa} + \mathrm{preAUT} \xrightarrow{k128} \mathrm{PKAa} + \mathrm{AUT}$$

$$\varnothing \xrightarrow{k129} \mathrm{ATG5}$$

$$\mathrm{ATG5t} \xrightarrow{k130} \varnothing$$

$$\mathrm{ATG5} \xrightarrow{k131} \varnothing$$

2 Revised reactions

The calculations were carried out in $\frac{mol}{cm^3}$, thus the rate coefficients values for the first and second order reactions are taken in $\frac{1}{s}$ and $\frac{cm^3}{mol*s}$ units, respectively.

$$\mathrm{DS} + \mathrm{P53} \xrightarrow{k1} \mathrm{P53A} + \mathrm{DS}$$

$$\mathrm{P53A} \xrightarrow{k3} \mathrm{MDM2} + \mathrm{P53A}$$

$$\mathrm{MDM2} \xrightarrow{k5} \varnothing$$

$$\mathrm{MDM2} + \mathrm{P53A} \xrightarrow{k7} \mathrm{MDM2}$$

$$\mathrm{P53A} + \mathrm{BAX} \xrightarrow{k9} \mathrm{BAXA} + \mathrm{P53A}$$

$$\mathrm{P53A} \xrightarrow{k11} \mathrm{P53A} + \mathrm{BAX}$$

$$\mathrm{P53A} + \mathrm{BCL2} \underset{k15}{\overset{k13}{\rightleftharpoons}} \mathrm{P53A\_BCL2}$$

$$\mathrm{BCL2} + \mathrm{BAX} \underset{k19}{\overset{k17}{\rightleftharpoons}} \mathrm{BCL2\_BAX}$$

$$\mathrm{BAXA} \xrightarrow{k21} \mathrm{BAX}$$

$$\mathrm{BAX} \xrightarrow{k23} \varnothing$$

$$\mathrm{BAXA} + \mathrm{CYTCM} \xrightarrow{k25} \mathrm{BAXA} + \mathrm{CYTC}$$

$$\mathrm{UVG} + \mathrm{BAX} \underset{k29}{\overset{k27}{\rightleftharpoons}} \mathrm{UVG\_BAX}$$

$$\mathrm{CALPAINA} + \mathrm{BID} \xrightarrow{k31} \mathrm{CALPAINA} + \mathrm{TBID}$$

$$\mathrm{TBID} \xrightarrow{k33} \varnothing$$

$$\varnothing \xrightarrow{k35} \mathrm{BID}$$

$$\mathrm{TBID} + \mathrm{CYTCM} \xrightarrow{k37} \mathrm{TBID} + \mathrm{CYTC}$$

$$\mathrm{CYTC} \xrightarrow{k39} \varnothing$$

$$\mathrm{CYTC} + \mathrm{PROCASP} \xrightarrow{k41} \mathrm{CYTC} + \mathrm{CASP}$$

$$\mathrm{CASP} + \mathrm{PROCASP} \xrightarrow{k43} 2\mathrm{CASP}$$

$$\mathrm{CASP} + \mathrm{BID} \xrightarrow{k45} \mathrm{TBID} + \mathrm{CASP}$$

$$\mathrm{DS} + \mathrm{DAPK} \xrightarrow{k47} \mathrm{DS} + \mathrm{DAPKA}$$

$$\mathrm{DAPKA} \xrightarrow{k49} \mathrm{DAPK}$$

$$\mathrm{P53A} + \mathrm{AMPKA} \xrightarrow{k51} \mathrm{P53A} + \mathrm{AMPK}$$

$$\mathrm{P53A} \xrightarrow{k53} \mathrm{P53A} + \mathrm{PUMA}$$

$$\mathrm{PUMA} + \mathrm{BCL2} \underset{k57}{\overset{k55}{\rightleftharpoons}} \mathrm{BCL2\_PUMA}$$

$$\mathrm{PUMA} \xrightarrow{k59} \varnothing$$

$$\mathrm{DAPKA + BEC1\_BCL2 \xrightarrow{k61} DAPKA + BEC1 + BCL2}$$
$$\mathrm{TG + SERCA \underset{k65}{\overset{k63}{\rightleftharpoons}} TG\_SERCA}$$
$$\mathrm{CA2IC + SERCA \xrightarrow{k67} CA2ER + SERCA}$$
$$\mathrm{CCH + PLCE \xrightarrow{k69} PLCEA}$$
$$\mathrm{EPACA + PLCE \xrightarrow{k71} EPACA + PLCEA}$$
$$\mathrm{PLCEA \xrightarrow{k73} PLCE}$$
$$\mathrm{PLCEA + PIP2 \xrightarrow{k75} PLCEA + IP3}$$
$$\mathrm{IP3R + IP3 \underset{k79}{\overset{k77}{\rightleftharpoons}} IP3R\_IP3}$$
$$\mathrm{CA2ER + IP3R_{IP3} \xrightarrow{k81} CA2IC + IP3R\_IP3}$$
$$\mathrm{IP3R\_IP3 + PHAG \xrightarrow{k83} IP3R\_IP3}$$
$$\mathrm{IP3 \xrightarrow{k85} PIP2}$$
$$\mathrm{CA2IC + CAMKKB \xrightarrow{k87} CA2IC + CAMKKBA}$$
$$\mathrm{AMPK + CAMKKBA \xrightarrow{k89} AMPKA + CAMKKBA}$$
$$\mathrm{CA2IC + CALPAIN \xrightarrow{k91} CA2IC + CALPAINA}$$
$$\mathrm{CALPAINA \xrightarrow{k93} CALPAIN}$$
$$\mathrm{CALPAINA + GA \xrightarrow{k95} GAA + CALPAINA}$$
$$\mathrm{AC + GAA \underset{k99}{\overset{k97}{\rightleftharpoons}} AC\_GAA}$$
$$\mathrm{AC_{GAA} \xrightarrow{k101} AC_{GAA} + CAMP}$$
$$\mathrm{CAMP \xrightarrow{k103} \varnothing}$$
$$\mathrm{CAMP + EPAC \xrightarrow{k105} CAMP + EPACA}$$
$$\mathrm{EPACA \xrightarrow{k107} EPAC}$$
$$\mathrm{GAA \xrightarrow{k109} GA}$$
$$\mathrm{GABC + GPCRA \xrightarrow{k111} GAA + GBC + GPCRA}$$
$$\mathrm{GA + GBC \xrightarrow{k113} GABC}$$
$$\mathrm{RAP + MTOR \underset{k117}{\overset{k115}{\rightleftharpoons}} MTOR\_RAP}$$
$$\mathrm{RAP + MTORA \underset{k121}{\overset{k119}{\rightleftharpoons}} MTOR\_RAP}$$
$$\mathrm{AMPKA + MTORA \xrightarrow{k123} AMPKA + MTOR}$$
$$\mathrm{MTORA \xrightarrow{k125} MTOR}$$
$$\mathrm{MTORA + AMPKA \xrightarrow{k127} MTORA + AMPK}$$
$$\mathrm{ULKA + MTORA \xrightarrow{k129} MTORA + ULK}$$

$$\mathrm{ULKA} + \mathrm{MTORA} \xrightarrow{k131} \mathrm{MTOR} + \mathrm{ULKA}$$
$$\mathrm{AMPKA} + \mathrm{TSC} \xrightarrow{k133} \mathrm{AMPKA} + \mathrm{TSCA}$$
$$\mathrm{RHEBA} + \mathrm{TSCA} \xrightarrow{k135} \mathrm{RHEB} + \mathrm{TSCA}$$
$$\mathrm{RHEBA} + \mathrm{MTOR} \xrightarrow{k137} \mathrm{RHEBA} + \mathrm{MTORA}$$
$$\mathrm{RHEB} \xrightarrow{k139} \mathrm{RHEBA}$$
$$\mathrm{AKTA} \xrightarrow{k141} \mathrm{AKT}$$
$$\mathrm{AKTA} + \mathrm{TSCA} \xrightarrow{k143} \mathrm{AKTA} + \mathrm{TSC}$$
$$\mathrm{ATG5T} + \mathrm{BCL2} \underset{k147}{\overset{k145}{\rightleftharpoons}} \mathrm{ATG5\_BCL2}$$
$$\mathrm{CALPAINA} + \mathrm{ATG5} \xrightarrow{k149} \mathrm{CALPAINA} + \mathrm{ATG5T}$$
$$\mathrm{CALPAINA} + \mathrm{BEC1} \xrightarrow{k151} \mathrm{CALPAINA}$$
$$\mathrm{IP3R} + \mathrm{BCL2} \underset{k155}{\overset{k153}{\rightleftharpoons}} \mathrm{IP3R\_BCL2}$$
$$\mathrm{BEC1} + \mathrm{BCL2} \underset{k159}{\overset{k157}{\rightleftharpoons}} \mathrm{BEC1\_BCL2}$$
$$\mathrm{ULKA} \xrightarrow{k161} \mathrm{ULKA} + \mathrm{PHAG}$$
$$\mathrm{AMPKA} + \mathrm{ULK} \xrightarrow{k163} \mathrm{AMPKA} + \mathrm{ULKA}$$
$$\mathrm{AMPK} + \mathrm{NS} \xrightarrow{k165} \mathrm{AMPKA} + \mathrm{NS}$$
$$\mathrm{AMPKA} \xrightarrow{k167} \mathrm{AMPK}$$
$$\mathrm{ULKA} \xrightarrow{k169} \mathrm{ULK}$$
$$\mathrm{AMPKA} + \mathrm{ULKA} \xrightarrow{k171} \mathrm{AMPK} + \mathrm{ULKA}$$
$$\mathrm{PHAG} + \mathrm{BEC1} \xrightarrow{k173} \mathrm{PREAUT} + \mathrm{BEC1}$$
$$\mathrm{PHAG} + \mathrm{BEC1\_UVG} \xrightarrow{k175} \mathrm{PREAUT} + \mathrm{BEC1\_UVG}$$
$$\mathrm{BEC1A} + \mathrm{UVG} \underset{k179}{\overset{k177}{\rightleftharpoons}} \mathrm{BEC1\_UVG}$$
$$\varnothing \xrightarrow{k181} \mathrm{BEC1}$$
$$\mathrm{PHAG} \xrightarrow{k183} \varnothing$$
$$\mathrm{ATG5} + \mathrm{PHAG} \xrightarrow{k185} \mathrm{ATG5} + \mathrm{AUT}$$
$$\mathrm{AUT} \xrightarrow{k187} \varnothing$$
$$\mathrm{AUT} + \mathrm{NS} \xrightarrow{k189} \mathrm{AUT}$$
$$\mathrm{AUT} + \mathrm{DS} \xrightarrow{k191} \mathrm{AUT}$$
$$\varnothing \xrightarrow{k193} \mathrm{PHAG}$$
$$\mathrm{PREAUT} \xrightarrow{k195} \varnothing$$
$$\mathrm{P53A} \xrightarrow{k197} \mathrm{P53A} + \mathrm{DRAM}$$

$$\mathrm{DRAM} \xrightarrow{k199} \varnothing$$

$$\mathrm{DRAM} + \mathrm{PREAUT} \xrightarrow{k201} \mathrm{DRAM} + \mathrm{AUT}$$

$$\varnothing \xrightarrow{k203} \mathrm{ATG5}$$

$$\mathrm{ATG5T} \xrightarrow{k205} \varnothing$$

$$\mathrm{ATG5} \xrightarrow{k207} \varnothing$$

$$\mathrm{CAMP} + \mathrm{PKA} \xrightarrow{k209} \mathrm{PKAA} + \mathrm{CAMP}$$

$$\mathrm{PKAA} + \mathrm{PHAG} \xrightarrow{k211} \mathrm{PKAA}$$

$$\mathrm{PKAA} \xrightarrow{k213} \mathrm{PKA}$$

$$\mathrm{PKC\_CA2IC} + \mathrm{PLCEA} \xrightarrow{k215} \mathrm{PLCE} + \mathrm{PKC\_CA2IC}$$

$$\mathrm{PKC} + \mathrm{CA2IC} \xrightarrow{k217} \mathrm{PKC}_{\mathrm{CA2IC}}$$

$$\mathrm{BEC1} + \mathrm{ULKA} \xrightarrow{k219} \mathrm{BEC1A} + \mathrm{ULKA}$$

$$\mathrm{INSULIN} + \mathrm{PI3K} \xrightarrow{k221} \mathrm{PI3KA} + \mathrm{INSULIN}$$

$$\mathrm{PI3KA} \xrightarrow{k223} \mathrm{PI3K}$$

$$\mathrm{PI3KA} + \mathrm{AKT} \xrightarrow{k225} \mathrm{AKTA} + \mathrm{PI3K}$$

Table S1: List of the species found in the models, and their full names

**Short Notation**	**Full Name**
AMPK	AMP-activated protein kinase
nS	Nuclear Stress
AMPKa	Active AMP-activated protein kinase
mTORa	Active mammalian target of rapamycin
mTOR	mammalian target of rapamycin
mTOR_ULK	mTOR ULK complex
ULKa	Active Unc-51 like autophagy activating kinase
ULK	Unc-51 like autophagy activating kinase
phag	Phagophore
TSC	Tuberous sclerosis complex
TSCa	Active tuberous sclerosis complex
RHEBa	Active Ras homolog enriched in brain
RHEB	Ras homolog enriched in brain
BEC1	Beclin 1
preAUT	Pre-autophagosome
BEC1_UVG	Beclin 1 UVRAG complex
UVG	UV radiation resistance-associated gene
BEC1c	Cytosolic Beclin 1
cytc	Cytochrome c

Table S1: List of the species found in the models, and their full names

Short Notation	Full Name
ATG5	Autophagy-related protein 5
AUT	Autophagosome
eS	ER Stress
PERK	Protein kinase R-like endoplasmic reticulum kinase
PERKa	Active PERK
ATF4a	Active Activating Transcription Factor 4
ATF4	Activating Transcription Factor 4
dS	DNA Stress
DAPK	Death-associated protein kinase
DAPKa	Active death-associated protein kinase
EPACa	Active exchange protein directly activated by cAMP
PLCe	Phospholipase C epsilon
PLCea	Active phospholipase C epsilon
PIP2	Phosphatidylinositol 4,5-bisphosphate
IP3	Inositol trisphosphate
Ca2er	Endoplasmic reticulum calcium ion
IP3R_IP3	IP3 receptor IP3 complex
Ca2ic	Intracellular calcium
SERCA	Sarcoplasmic/endoplasmic reticulum calcium ATPase
Bax	Bcl-2-associated X protein
Baxa	Active Bcl-2-associated X protein
IP3R	IP3 receptor
IP2	Inositol bisphosphate
IP	Inositol phosphate
Ins	Insulin
CaMKKb	Calcium/calmodulin-dependent protein kinase kinase beta
CaMKKba	Active calcium/calmodulin-dependent protein kinase kinase b
BCL2	B-cell lymphoma 2
IP3R_BCL2	IP3 receptor BCL2 complex
BEC1_BCL2	Beclin 1 BCL2 complex
BCL2p	Phosphorylated BCL2
Calpain	Calpain
Calpaina	Active calpain
Ga	G protein alpha subunit
Gaa	Active G protein alpha subunit
ATG5t	truncated ATG5
ATG5_BCL2	ATG5 BCL2 complex
Bid	BH3 interacting-domain death agonist
tBid	Truncated Bid

Table S1: List of the species found in the models, and their full names

Short Notation	Full Name
AC	Adenylate cyclase
AC_Gaa	Adenylate cyclase G protein alpha subunit complex
cAMP	Cyclic adenosine monophosphate
EPAC	Exchange protein directly activated by cAMP
G	G protein
AC_Ga	Adenylate cyclase G protein alpha subunit complex
Gabg	G protein alpha, beta, gamma subunits
GPCRa	Active G protein-coupled receptor
Gbg	G protein beta and gamma subunits
BCL2_Bax	BCL2 Bax complex
p53m	Mitochondrial p53
UVG_Bax	UVG bound to Bax
caspase	Procaspase
caspasea	Cleaved caspase
JNKa	Active c-Jun N-terminal kinase
p53c	Cytosolic p53
p53a	Active p53
Mdm2	Mouse double minute 2 homolog
p53_BCL2	p53 BCL2 complex
DRAM	DNA damage-regulated autophagy modulator
PUMA	p53 upregulated modulator of apoptosis
PUMA_BCL2	PUMA BCL2 complex
TFEBi	Inactive transcription factor EB
TFEB	Transcription factor EB
PI3K	Phosphoinositide 3-kinase
PI3Ka	Active phosphoinositide 3-kinase
AKT	Protein kinase B
AKTa	Active protein kinase B
JNK	c-Jun N-terminal kinase
MAPK15	Mitogen-activated protein kinase 15
MAPK15a	Active mitogen-activated protein kinase 15
PKA	Protein kinase A
PKAa	Active protein kinase A

3 Initial protein concentrations

Table S2: Initial species concentration ranges in nM

species	min	max	source
AC	100	400	[1, 2]
AC_Ga	0	100	assumed
AC_Gaa	0	100	assumed
AKT	0	100	[3, 4]
AKTa	50	200	[3, 4]
AMPK	187.5	750	[5, 6]
AMPKa	0	100	[5, 6]
ATG5	100	400	[7]
ATG5_BCL2	0	100	assumed
ATG5t	15	60	assumed
AUT	0	100	[8]
BCL2	25	100	[9, 10]
BCL2_Bax	10	40	[9]
BCL2_PUMA	25	100	[9]
BEC1	50	200	[11]
BEC1_BCL2	0	100	assumed
BEC1_UVG	0	100	assumed
Bax	10	40	[9, 10]
Baxa	0	0	[9, 10]
Bid	10	40	[9, 10]
Ca2er	500	2000	[12, 13, 14]
Ca2ic	50	200	[12, 13, 14]
CaMKKb	50	200	[12]
CaMKKba	0	100	[12]
Calpain	10	40	[15]
Calpaina	0	100	[15]
DAPK	50	200	[11]
DAPKa	0	100	[11]
DRAM	0	100	[11]
EPAC	5	20	assumed
EPACa	0	100	assumed
GPCRa	5	20	[16]
Ga	5	20	[16, 17, 13]
Gaa	0	100	[16, 17, 13]
Gabc	0	100	[16, 17, 13]
Gbc	5	20	[16, 17, 13]
IP3	65	260	[12]
IP3R	5	20	[12]
IP3R_BCL2	0	100	assumed

IP3R_IP3	0	100	assumed
MDM2	0	100	[18, 19]
P53a_BCL2	0	100	assumed
PLCea	0	100	[12]
PLCe	5	20	[12, 14, 20]
PIP2	250	1000	[12]
PKA	75	300	[2]
PKAa	0	100	assumed
PUMA	0	100	assumed
RHEB	0	100	assumed
RHEBa	150	600	assumed
SERCA	0	100	[12]
TSC	112.5	450	[5, 6]
TSCa	0	100	[5, 6]
ULK	35	140	[8]
ULKa	0	100	assumed
UVG	50	200	assumed
UVG_Bax	0	100	assumed
cAMP	0	100	[1, 2]
casp	0	0	assumed
cytc	0	100	[9, 10]
cytcm	10	40	[9, 10]
mTOR	0	100	[5, 11]
mTORa	187.5	750	[5, 11]
mTORa_ULK	0	100	assumed
mTORa_ULKa	0	100	assumed
p53	12.5	50	[18, 19]
p53a	0	100	assumed
phag	0	100	assumed
preAUT	0	100	assumed
procasp	12	48	[9, 21]
tBid	0	100	[9]

References

[1] Iancu, R.V.; Jones, S.W.; Harvey, R.D. Compartmentation of camp signaling in cardiac myocytes: A computational study. *Biophys. J.* **2007**, *92*, 3317–3331.

[2] Nakano, T.; Doi, T.; Yoshimoto, J.; Doya, K. A kinetic model of dopamine- and calcium-dependent striatal synaptic plasticity. *PLoS Comput. Biol.* **2010**, *6*, e1000670.

[3] Shin, S.-Y.; Nguyen, L. Unveiling hidden dynamics of hippo signalling: A systems analysis. *Genes* **2016**, *7*, 44.

[4] Hat, B.; Kochańczyk, M.; Bogdał, M.N.; Lipniacki, T. Feedbacks, bifurcations, and cell fate decision-making in the p53 system. *PLOS Comput. Biol.* **2016**, *12*, e1004787.

[5] Pezze, P.D.; Sonntag, A.G.; Thien, A.; Prentzell, M.T.; Gödel, M.; Fischer, S.; Neumann-Haefelin, E.; Huber, T.B.; Baumeister, R.; Shanley, D.P.; et al. A dynamic network model of mtor signaling reveals tsc-independent mtorc2 regulation. *Sci. Signal.* **2012**, *5*, ra25.

[6] Varusai, T.M.; Nguyen, L.K. Dynamic modelling of the mtor signalling network reveals complex emergent behaviours conferred by deptor. *Sci. Rep.* **2018**, *8*, 643.

[7] Broadbent, D.G.; Barnaba, C.; Perez, G.I.; Schmidt, J.C. Quantitative analysis of autophagy reveals the role of atg9 and atg2 in autophagosome formation. *J. Cell Biol.* **2023**, *222*, e202210078.

[8] du Toit, A.; Hofmeyr, J.-H.S.; Gniadek, T.J.; Loos, B. Measuring autophagosome flux. *Autophagy* **2018**, *14*, 1060–1071.

[9] Bagci, E.Z.; Vodovotz, Y.; Billiar, T.R.; Ermentrout, G.B.; Bahar, I. Bistability in apoptosis: Roles of bax, bcl-2, and mitochondrial permeability transition pores. *Biophys. J.* **2006**, *90*, 1546–1559.

[10] Raychaudhuri, S.; Das, S. Low probability activation of bax/bak can induce selective killing of cancer cells by generating heterogeneity in apoptosis. *J. Healthc. Eng.* **2013**, *4*, 47–66.

[11] Tavassoly, I.; Parmar, J.; Shajahan-Haq, A.N.; Clarke, R.; Baumann, W.T.; Tyson, J.J. Dynamic modeling of the interaction between autophagy and apoptosis in mammalian cells. *CPT Pharmacometr. Syst. Pharmacol.* **2015**, *4*, 263–272.

[12] Dolan, A.T.; Diamond, S.L. Systems modeling of ca2+ homeostasis and mobilization in platelets mediated by ip3 and store-operated ca2+ entry. *Biophys. J.* **2014**, *106*, 2049–2060.

[13] Lemon, G.; Gibson, W.G.; Bennett, M.R. Metabotropic receptor activation, desensitization and sequestration—i: Modelling calcium and inositol 1,4,5-trisphosphate dynamics following receptor activation. *J. Theor. Biol.* **2003**, *223*, 93–111.

[14] Wang, J.; Huang, X.; Huang, W. A quantitative kinetic model for atp-induced intracellular oscillations. *J. Theor. Biol.* **2007**, *245*, 510–519.

[15] Ashraf, J.; Ahmad, J.; Ul-Haq, Z. *Deciphering the Role of PKC in Calpain-CAST System Through Formal Modeling Approach*; Springer International Publishing: Berlin/Heidelberg, Germany, 2019; pp. 60–71.

[16] Smith, B.; Hill, C.; Godfrey, E.L.; Rand, D.; van den Berg, H.; Thornton, S.; Hodgkin, M.; Davey, J.; Ladds, G. Dual positive and negative regulation of gpcr signaling by gtp hydrolysis. *Cell. Signal.* **2009**, *21*, 1151–1160.

[17] Tiveci, S.; Akın, A.; Çakır, T.; Saybaşılı, H.; Ülgen, K. Modelling of calcium dynamics in brain energy metabolism and alzheimer's disease. *Comput. Biol. Chem.* **2005**, *29*, 151–162.

[18] Chong, K.H.; Samarasinghe, S.; Kulasiri, D. Mathematical modelling of p53 basal dynamics and dna damage response. *Math. Biosci.* **2015**, *259*, 27–42.

[19] Geva-Zatorsky, N.; Rosenfeld, N.; Itzkovitz, S.; Milo, R.; Sigal, A.; Dekel, E.; Yarnitzky, T.; Liron, Y.; Polak, P.; Lahav, G.; et al. Oscillations and variability in the p53 system. *Mol. Syst. Biol.* **2006**, *2*, 2006.0033.

[20] Eungdamrong, N.J.; Iyengar, R. Compartment-specific feedback loop and regulated trafficking can result in sustained activation of ras at the golgi. *Biophys. J.* **2007**, *92*, 808–815.

[21] Neumann, L.; Pforr, C.; Beaudouin, J.; Pappa, A.; Fricker, N.; Krammer, P.H.; Lavrik, I.N.; Eils, R. Dynamics within the cd95 death-inducing signaling complex decide life and death of cells. *Mol. Syst. Biol.* **2010**, *6*, 352.
