# Supplementary material for: Basal State Calibration of a Chemical Reaction Network Model for Autophagy
Source: Int J Mol Sci. 2024 Oct 21;25(20):11316. doi: 10.3390/ijms252011316 (PMC11508741; doi:10.3390/ijms252011316)
Supplement: Supplementary file 1 [file ijms-25-11316-s001.zip › supplement/supplementary_information_2.pdf]

Supplementary Table 2.: Initial and Optimized Rate Parameters (logarithmic scale, base 10) and Posterior Uncertainty Parameters (fff) with Uncertainty Factors for all Reactions

#	Reaction	$\log_{10}k_{ini}$	f_{prior}	$\log_{10}k_{opt}$	$f_{posterior}$	$10^{f_{posterior.}}$
73	ATG5T+BCL2⟶ATG5_BCL2	6.50	4.00	4.79	0.13	1.35
43	IP3⟶PIP2	-3.55	4.00	-5.15	0.14	1.37
104	ATG5⟶REF	-4.55	4.00	-4.51	0.19	1.55
102	REF⟶ATG5	-15.55	4.00	-14.23	0.20	1.57
109	PKC+CA2IC⟶PKC_CA2IC	5.44	4.00	4.17	0.26	1.81
10	BCL2_BAX⟶BCL2+BAX	-3.50	4.00	-5.76	0.26	1.84
63	MTORA⟶MTOR	-3.55	4.00	-3.45	0.60	3.98
71	AKTA⟶AKT	-3.53	4.00	-6.30	0.63	4.25
54	EPACA⟶EPAC	-3.50	4.00	-4.83	0.63	4.26
18	REF⟶BID	-17.59	4.00	-16.63	0.65	4.49
30	PUMA⟶REF	-3.92	4.00	-4.99	0.73	5.36
29	BCL2_PUMA⟶PUMA+BCL2	-3.08	4.00	-3.34	0.87	7.36
69	RHEBA+MTOR⟶RHEBA+MTORA	6.45	4.00	8.16	0.88	7.65
28	PUMA+BCL2⟶BCL2_PUMA	7.22	4.00	7.91	0.89	7.70
9	BCL2+BAX⟶BCL2_BAX	6.54	4.00	3.18	0.94	8.77
8	P53A_BCL2⟶P53A+BCL2	-6.78	4.00	-6.32	0.97	9.32
44	CA2IC+CAMKKB⟶CA2IC+CAMKKBA	5.44	4.00	3.12	0.99	9.73
34	CA2IC+SERCA⟶CA2ER+SERCA	7.01	4.00	3.47	1.00	10.0
45	AMPK+CAMKKBA⟶AMPKA+CAMKKBA	6.44	4.00	6.05	1.00	10.0
57	GA+GBC⟶GABC	5.70	4.00	6.03	1.04	10.9
38	PLCEA+PIP2⟶PLCEA+IP3	8.52	4.00	8.74	1.04	11.0
85	ULKA⟶ULK	-4.55	4.00	-0.55	1.04	11.1
65	ULKA+MTORA=MTORA+ULK	6.42	4.00	8.21	1.05	11.1
56	GABC+GPCRA⟶GAA+GBC+GPCRA	8.54	4.00	7.61	1.07	11.7
6	P53A⟶P53A+BAX	8.44	4.00	6.41	1.16	14.3
36	EPACA+PLCE⟶EPACA+PLCEA	8.40	4.00	7.90	1.16	14.3
31	DAPKA+BEC1_BCL2⟶DAPKA+BEC1+BCL2	8.71	4.00	10.15	1.17	14.7
4	MDM2+P53A⟶MDM2	6.42	4.00	9.08	1.17	14.7
14	UVG+BAX⟶UVG_BAX	6.41	4.00	6.71	1.18	15.2
55	GAA⟶GA	-3.56	4.00	-0.25	1.20	16.0
7	P53A+BCL2⟶P53A_BCL2	6.43	4.00	8.60	1.21	16.2
62	AMPKA+MTORA⟶AMPKA+MTOR	6.44	4.00	7.78	1.24	17.3
5	P53A+BAX⟶BAXA+P53A	8.44	4.00	9.80	1.25	17.8
41	CA2ER+IP3R_IP3⟶CA2IC+IP3R_IP3	8.42	4.00	6.69	1.26	18.3
48	CALPAINA+GA⟶GAA+CALPAINA	6.51	4.00	7.58	1.27	18.5
94	AUT⟶REF	-3.56	4.00	-4.70	1.27	18.6
108	PKC_CA2IC+PLCEA⟶PLCE+PKC_CA2IC	8.40	4.00	8.59	1.27	18.8
12	BAX⟶REF	-3.55	4.00	-0.37	1.27	18.8
80	BEC1_BCL2⟶BEC1+BCL2	-3.59	4.00	-0.95	1.28	19.3
52	CAMP⟶REF	-2.86	4.00	-2.69	1.29	19.6
100	DRAM⟶REF	-3.57	4.00	-6.78	1.30	19.7
49	AC+GAA⟶AC_GAA	6.37	4.00	9.05	1.30	19.8
15	UVG_BAX⟶UVG+BAX	-4.59	4.00	-1.92	1.30	19.9
40	IP3R_IP3⟶IP3R+IP3	-3.59	4.00	-2.12	1.30	20.0

106	PKAA+PHAG⟶PKAA	5.64	4.00	8.52	1.31	20.4
84	AMPKA⟶AMPK	-3.58	4.00	-6.39	1.31	20.4
88	PHAG+BEC1_UVG⟶PREAUT+BEC1_UVG	6.44	4.00	9.40	1.32	21.1
37	PLCEA⟶PLCE	-5.49	4.00	-1.97	1.34	21.8
53	CAMP+EPAC⟶CAMP+EPACA	6.54	4.00	6.91	1.34	21.9
51	AC_GAA⟶AC_GAA+CAMP	-3.48	4.00	-5.28	1.35	22.2
11	BAXA⟶BAX	-2.85	4.00	-0.52	1.36	22.7
87	PHAG+BEC1⟶PREAUT+BEC1	5.74	4.00	5.43	1.36	22.7
107	PKAA⟶PKA	-2.89	4.00	-5.18	1.36	23.0
3	MDM2⟶REF	-3.52	4.00	-2.67	1.36	23.1
101	DRAM+PREAUT⟶DRAM+AUT	6.44	4.00	6.74	1.37	23.3
47	CALPAINA⟶CALPAIN	-3.57	4.00	-0.09	1.38	24.1
103	ATG5T⟶REF	-3.57	4.00	-6.79	1.39	24.3
68	RHEBA+TSCA⟶RHEB+TSCA	7.45	4.00	3.72	1.39	24.5
76	CALPAINA+BEC1⟶CALPAINA	5.97	4.00	5.07	1.40	25.4
22	CASP+PROCASP⟶CASP+CASP	6.44	4.00	6.43	1.42	26.0
81	ULKA⟶ULKA+PHAG	-3.56	4.00	-7.10	1.42	26.1
70	RHEB⟶RHEBA	-3.60	4.00	-7.04	1.42	26.2
66	ULKA+MTORA=MTOR+ULKA	6.42	4.00	5.29	1.42	26.6
89	BEC1A+UVG⟶BEC1_UVG	6.44	4.00	7.14	1.43	26.7
16	CALPAINA+BID⟶CALPAINA+TBID	6.45	4.00	7.82	1.44	27.5
98	PREAUT⟶REF	-4.56	4.00	-7.23	1.44	27.8
75	CALPAINA+ATG5⟶CALPAINA+ATG5T	6.52	4.00	6.35	1.46	28.5
2	P53A⟶MDM2+P53A	-3.63	4.00	-4.90	1.46	28.6
91	REF⟶BEC1	-17.51	4.00	-18.95	1.46	28.7
26	P53A+AMPKA⟶P53A+AMPK	6.42	4.00	5.83	1.46	28.7
46	CA2IC+CALPAIN⟶CA2IC+CALPAINA	6.50	4.00	6.74	1.48	30.5
110	BEC1+ULKA⟶BEC1A+ULKA	6.43	4.00	3.61	1.49	31.1
72	AKTA+TSCA⟶AKTA+TSC	8.41	4.00	9.81	1.49	31.1
39	IP3R+IP3⟶IP3R_IP3	6.45	4.00	6.46	1.50	31.9
99	P53A⟶P53A+DRAM	-3.52	4.00	-3.19	1.51	32.2
74	ATG5_BCL2⟶ATG5T+BCL2	-7.11	4.00	-8.67	1.52	33.2
86	AMPKA+ULKA⟶AMPK+ULKA	6.43	4.00	5.22	1.53	33.8
77	IP3R+BCL2⟶IP3R_BCL2	6.47	4.00	5.40	1.53	34.1
20	CYTC⟶REF	-3.56	4.00	-2.69	1.54	34.5
50	AC_GAA⟶AC+GAA	-3.60	4.00	-0.01	1.54	34.7
78	IP3R_BCL2⟶IP3R+BCL2	-3.60	4.00	-6.26	1.55	35.4
90	BEC1_UVG⟶BEC1A+UVG	-3.55	4.00	-3.43	1.56	36.3
93	ATG5+PHAG⟶ATG5+AUT	8.44	4.00	10.00	1.65	44.7
82	AMPKA+ULK⟶AMPKA+ULKA	6.44	4.00	3.60	1.67	46.7
17	TBID⟶REF	-3.59	4.00	-4.32	1.73	54.2
67	AMPKA+TSC⟶AMPKA+TSCA	6.45	4.00	4.26	1.78	60.1
25	DAPKA⟶DAPK	-3.53	4.00	-4.15	1.78	60.8
79	BEC1+BCL2⟶BEC1_BCL2	6.45	4.00	4.09	1.91	80.5
97	REF⟶PHAG	-19.55	4.00	-18.52	1.97	92.8
105	CAMP+PKA⟶PKAA+CAMP	6.94	4.00	6.19	1.98	94.6
92	PHAG⟶REF	-4.55	4.00	-5.53	1.99	97.7
27	P53A⟶P53A+PUMA	-3.85	4.00	-5.98	2.08	119.3

64	MTORA+AMPKA⟶MTORA+AMPK	6.44	4.00	4.59	2.08	121.4
42	IP3R_IP3+PHAG⟶IP3R_IP3	6.37	4.00	7.54	2.23	171.8
23	CASP+BID⟶TBID+CASP	6.44	4.00	6.51	2.39	246.6
13	BAXA+CYTCM⟶BAXA+CYTC	-3.56	4.00	-7.34	2.60	394.4
21	CYTC+PROCASP⟶CYTC+CASP	6.44	4.00	3.54	2.71	512.1
19	TBID+CYTCM⟶TBID+CYTC	-3.56	4.00	-3.23	4.43	26672.5
