# Supplementary figures and images for: Basal State Calibration of a Chemical Reaction Network Model for Autophagy
Source: Int J Mol Sci. 2024 Oct 21;25(20):11316. doi: 10.3390/ijms252011316 (PMC11508741; doi:10.3390/ijms252011316)

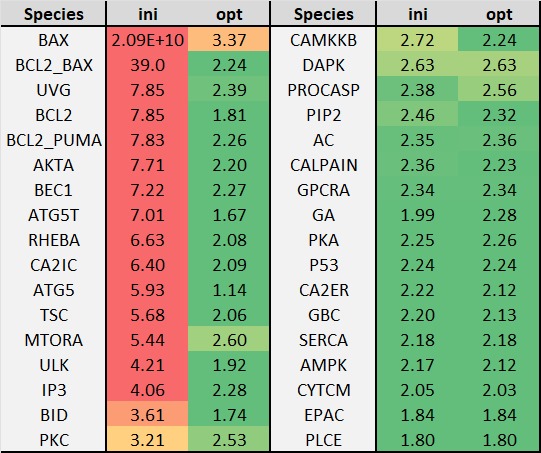

Supplement: Supplementary file 1 [file ijms-25-11316-s001.zip › figures/errortable.jpeg]

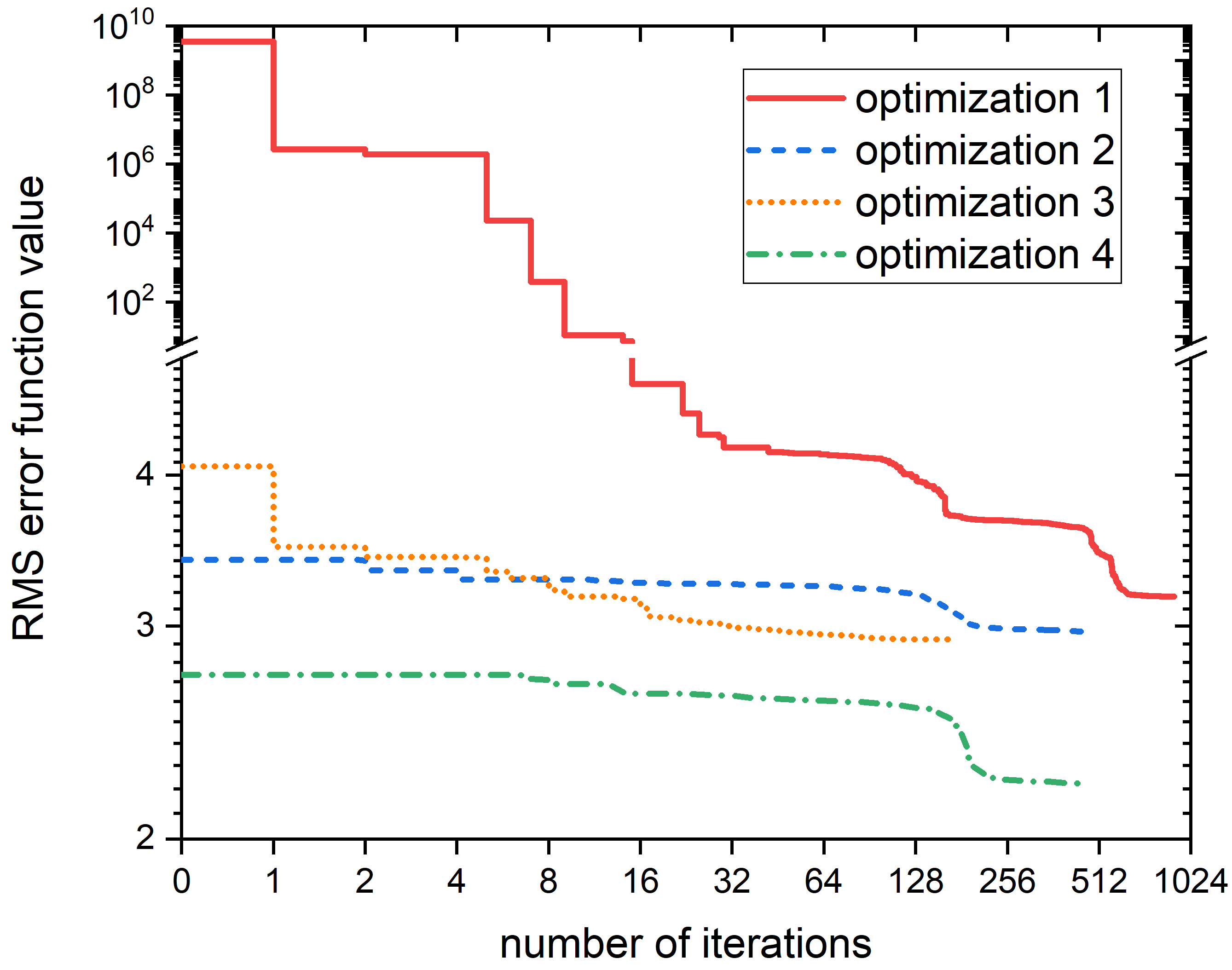

Supplement: Supplementary file 1 [file ijms-25-11316-s001.zip › figures/error_evolution.png]

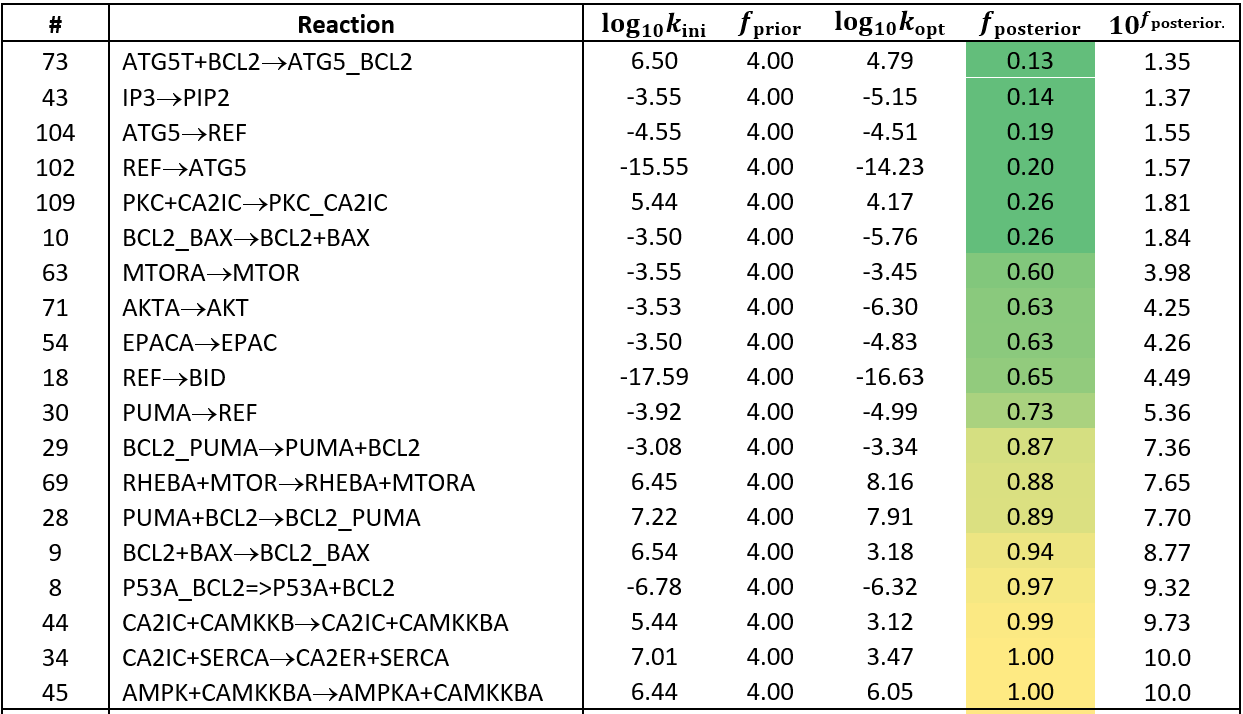

Supplement: Supplementary file 1 [file ijms-25-11316-s001.zip › figures/image.png]

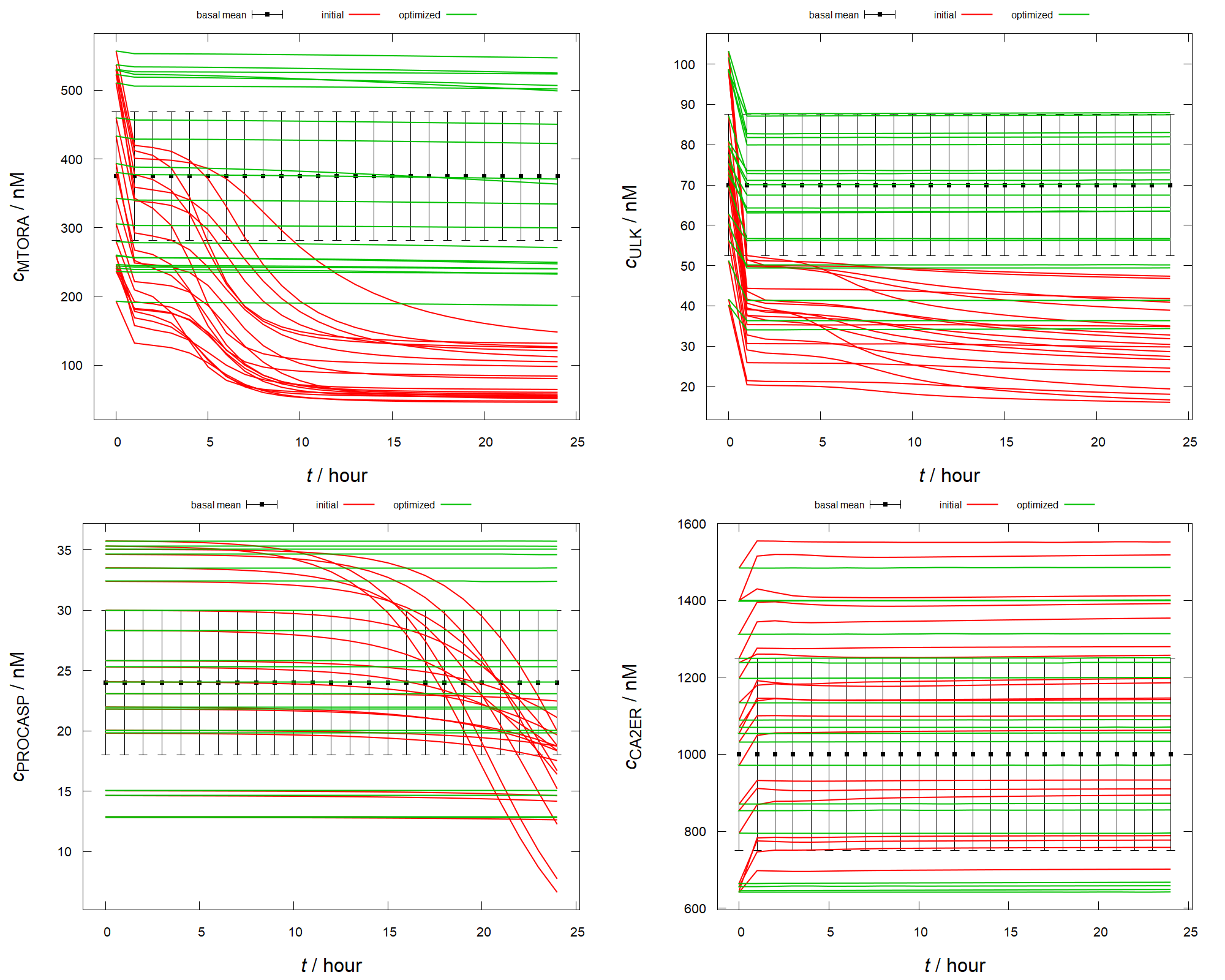

Supplement: Supplementary file 1 [file ijms-25-11316-s001.zip › figures/MTORA-ULK-PROCASP-CA2ER.png]

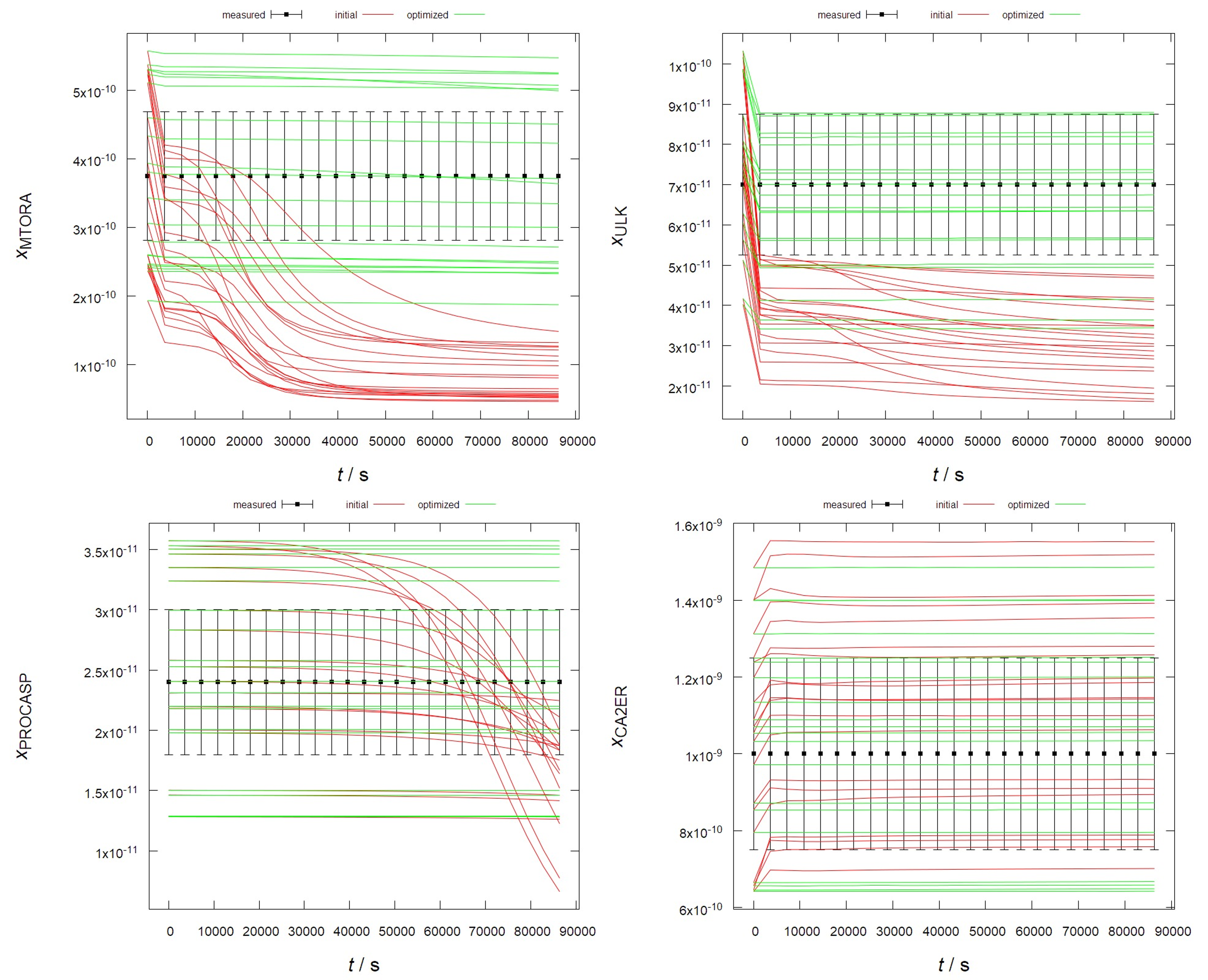

Supplement: Supplementary file 1 [file ijms-25-11316-s001.zip › figures/species.png]

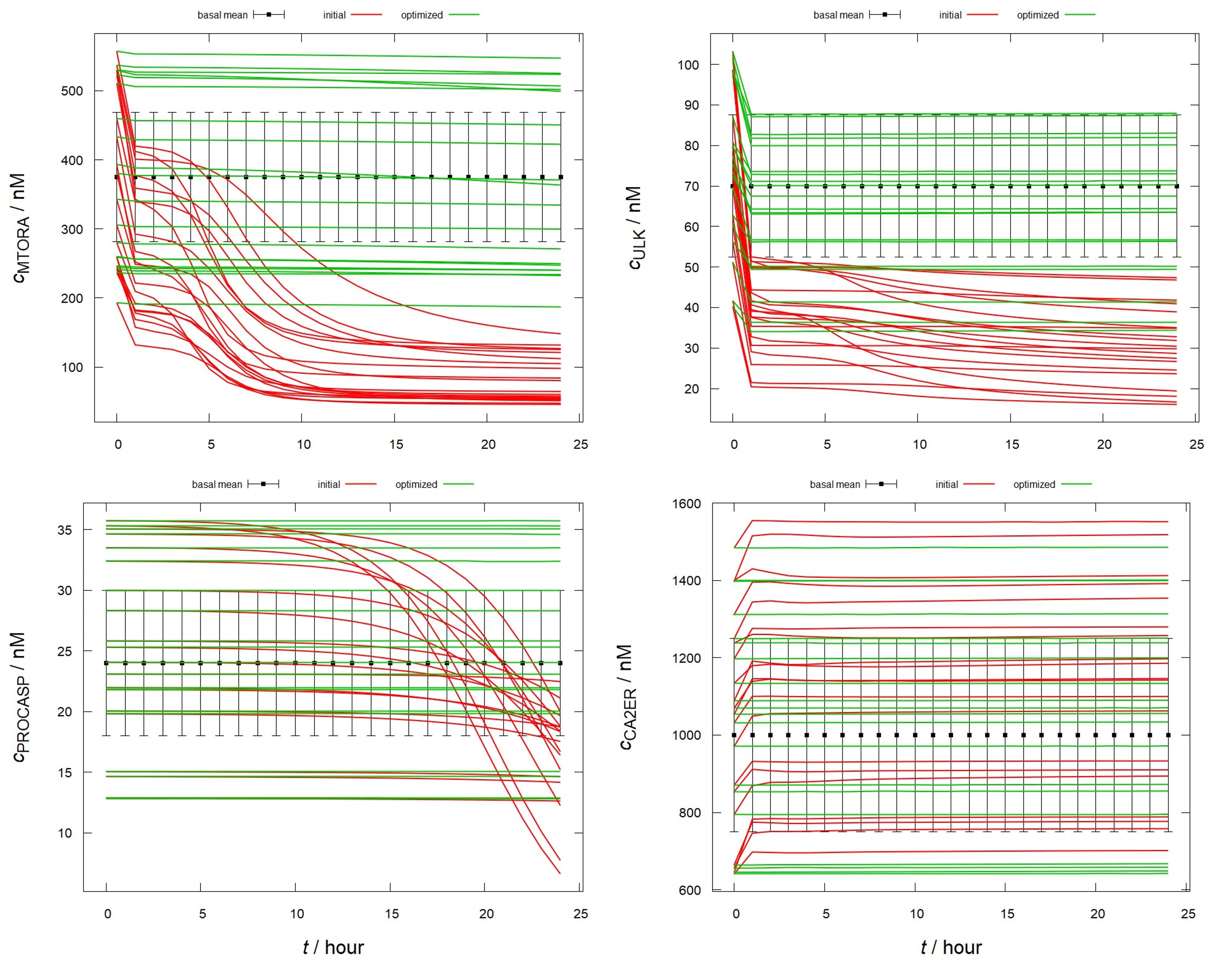

Supplement: Supplementary file 1 [file ijms-25-11316-s001.zip › figures/species_final.png]

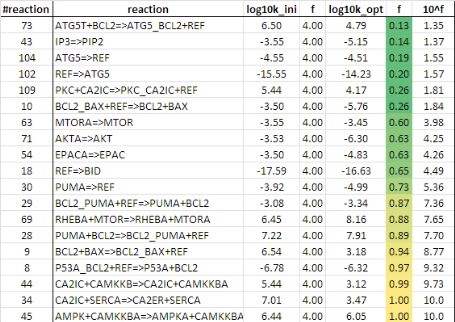

Supplement: Supplementary file 1 [file ijms-25-11316-s001.zip › figures/uncertainty.jpg]

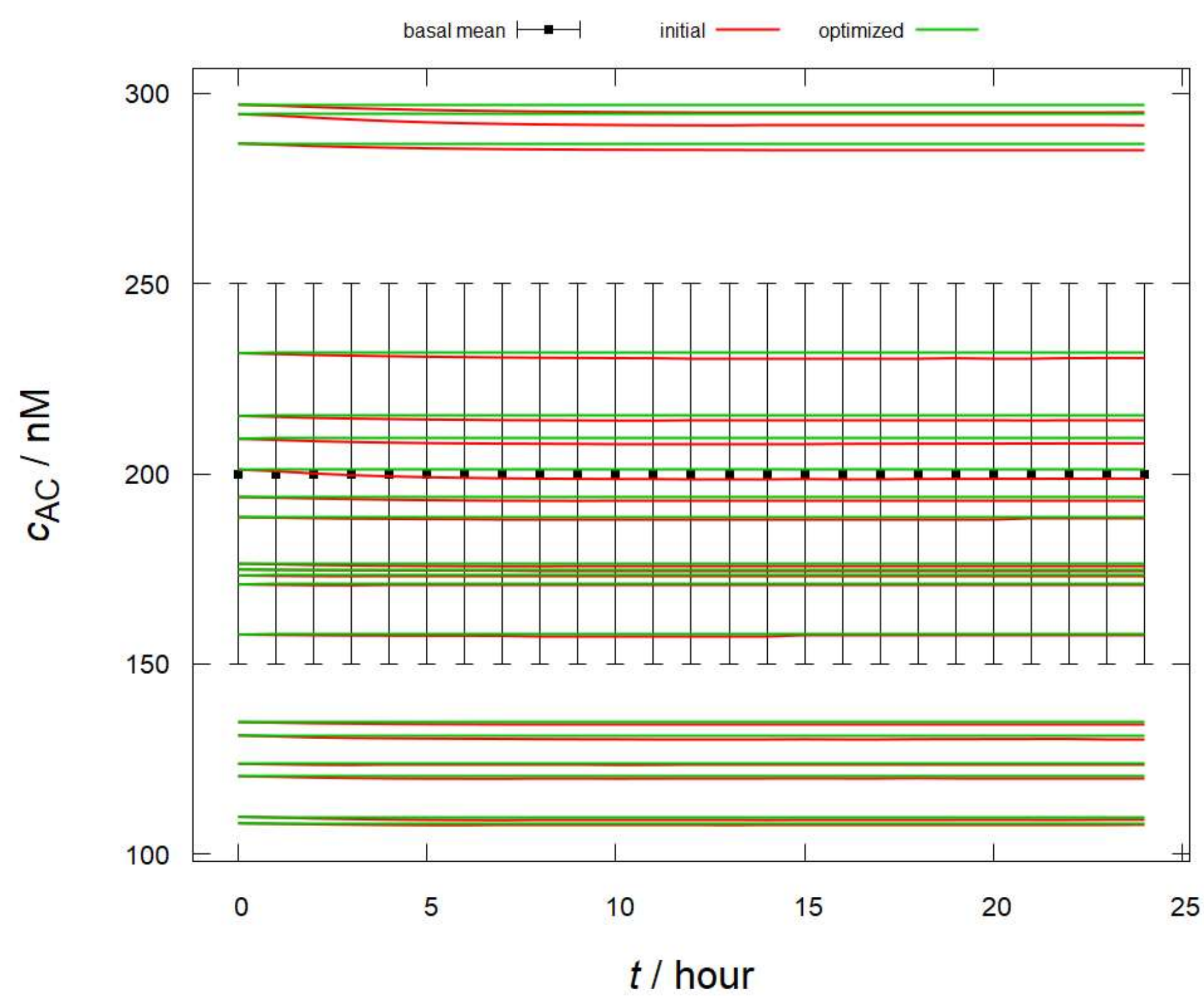

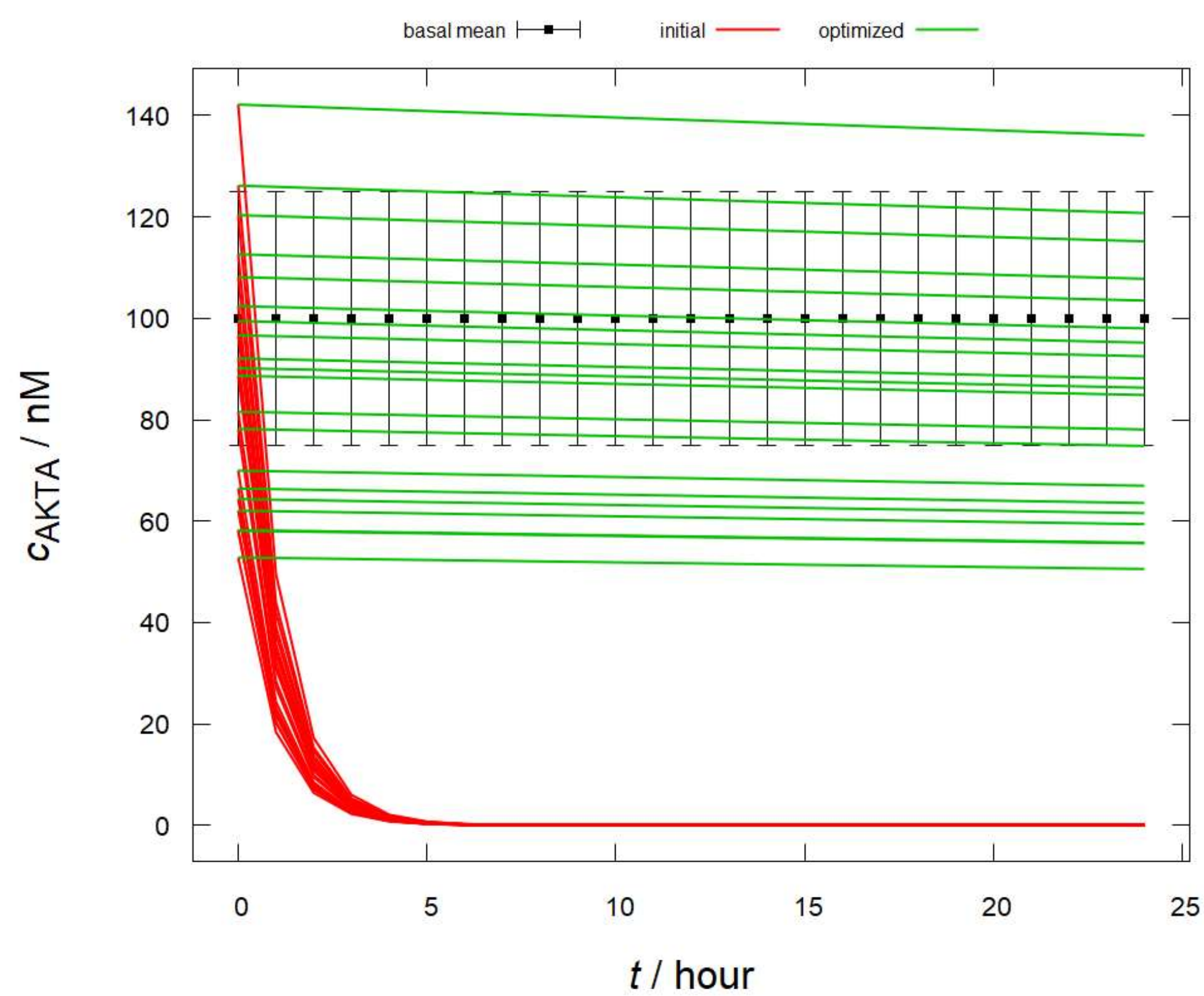

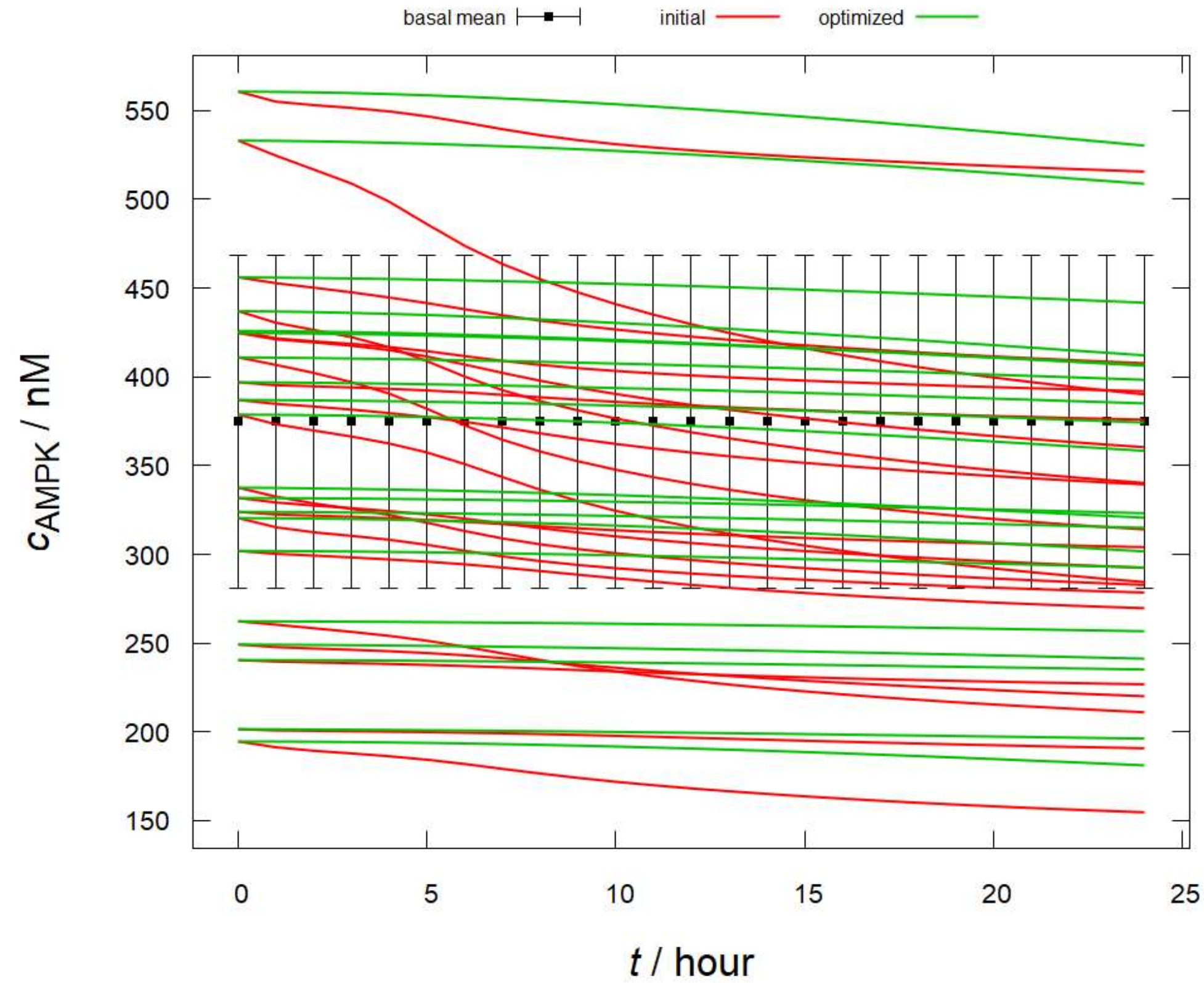

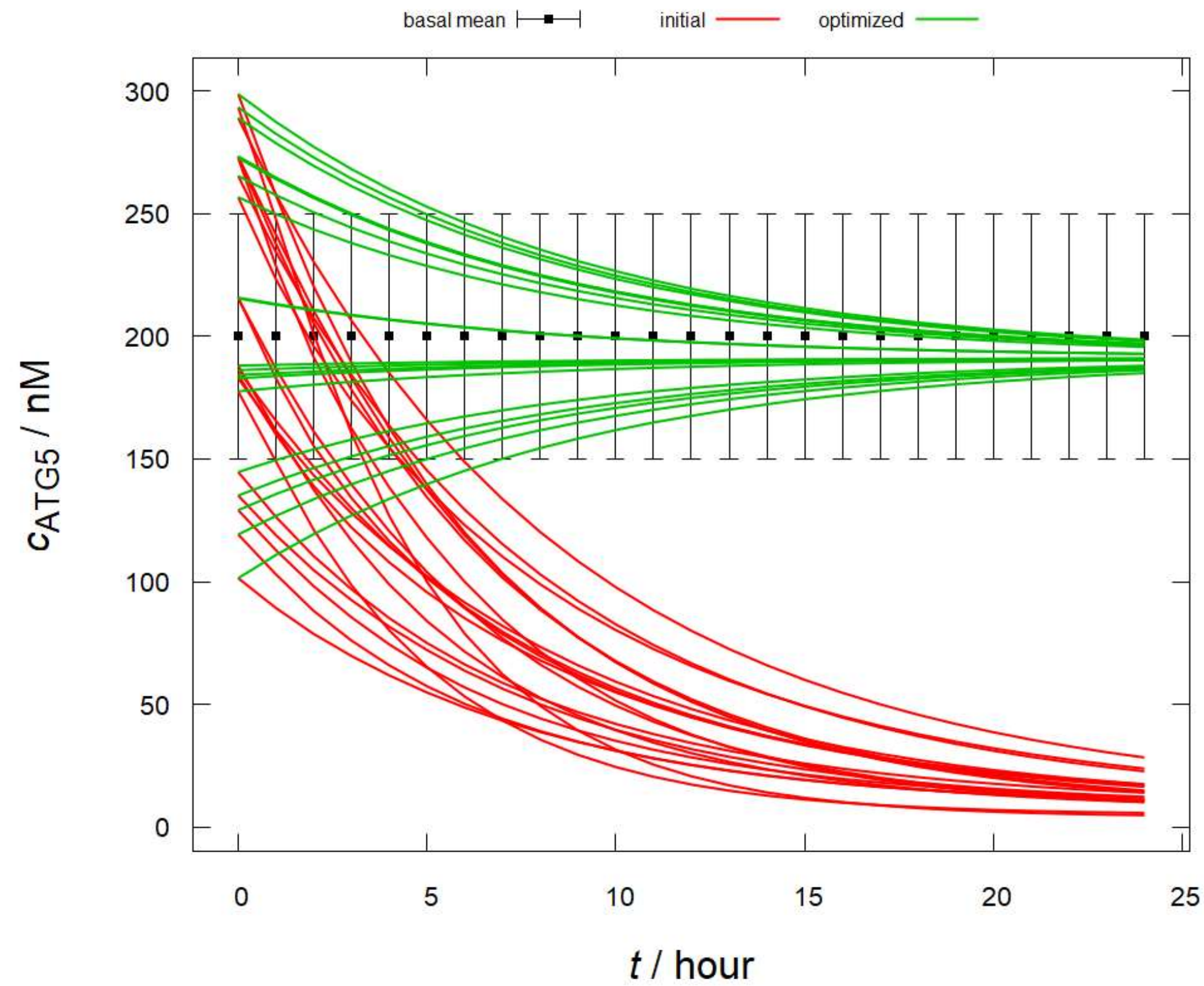

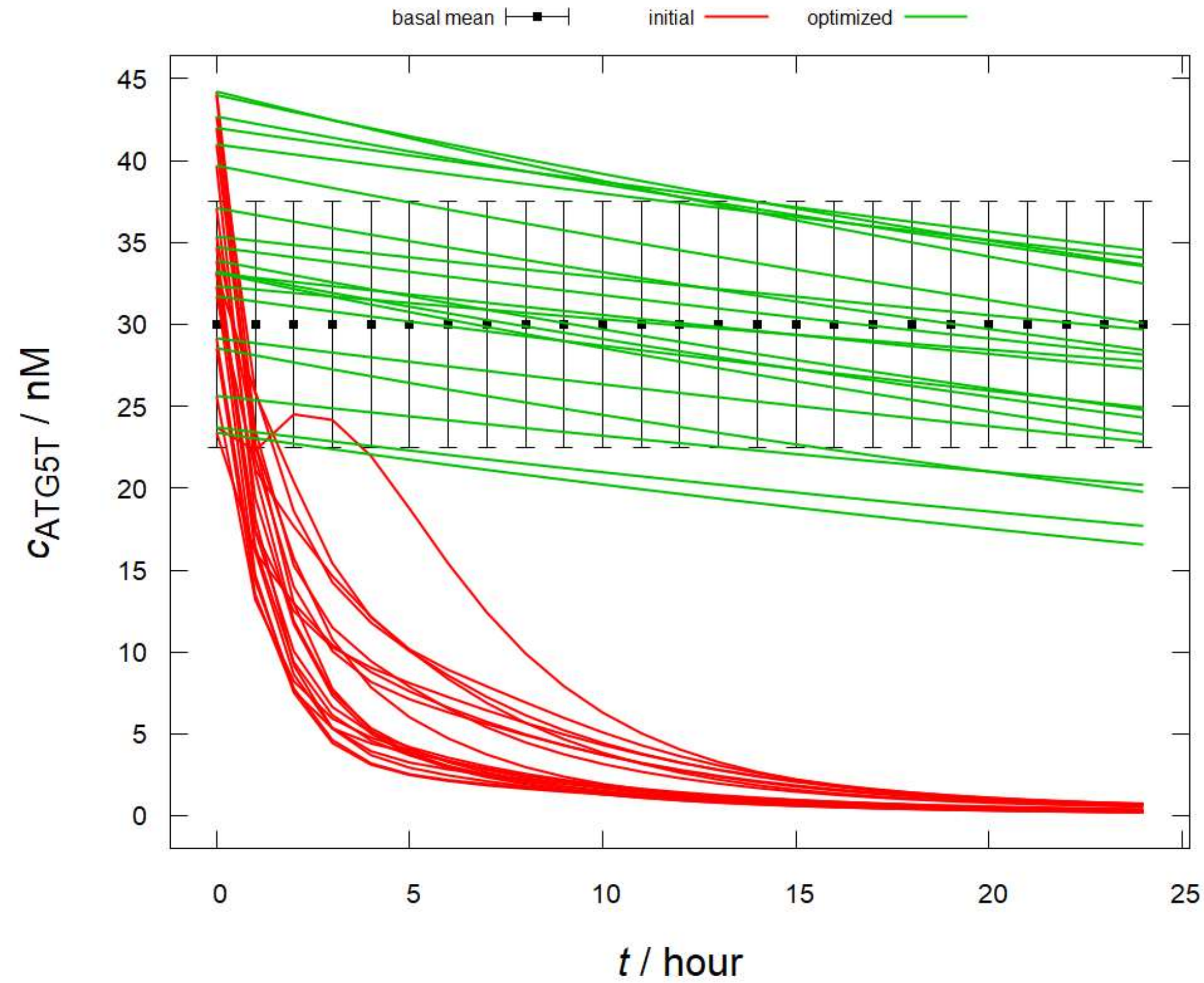

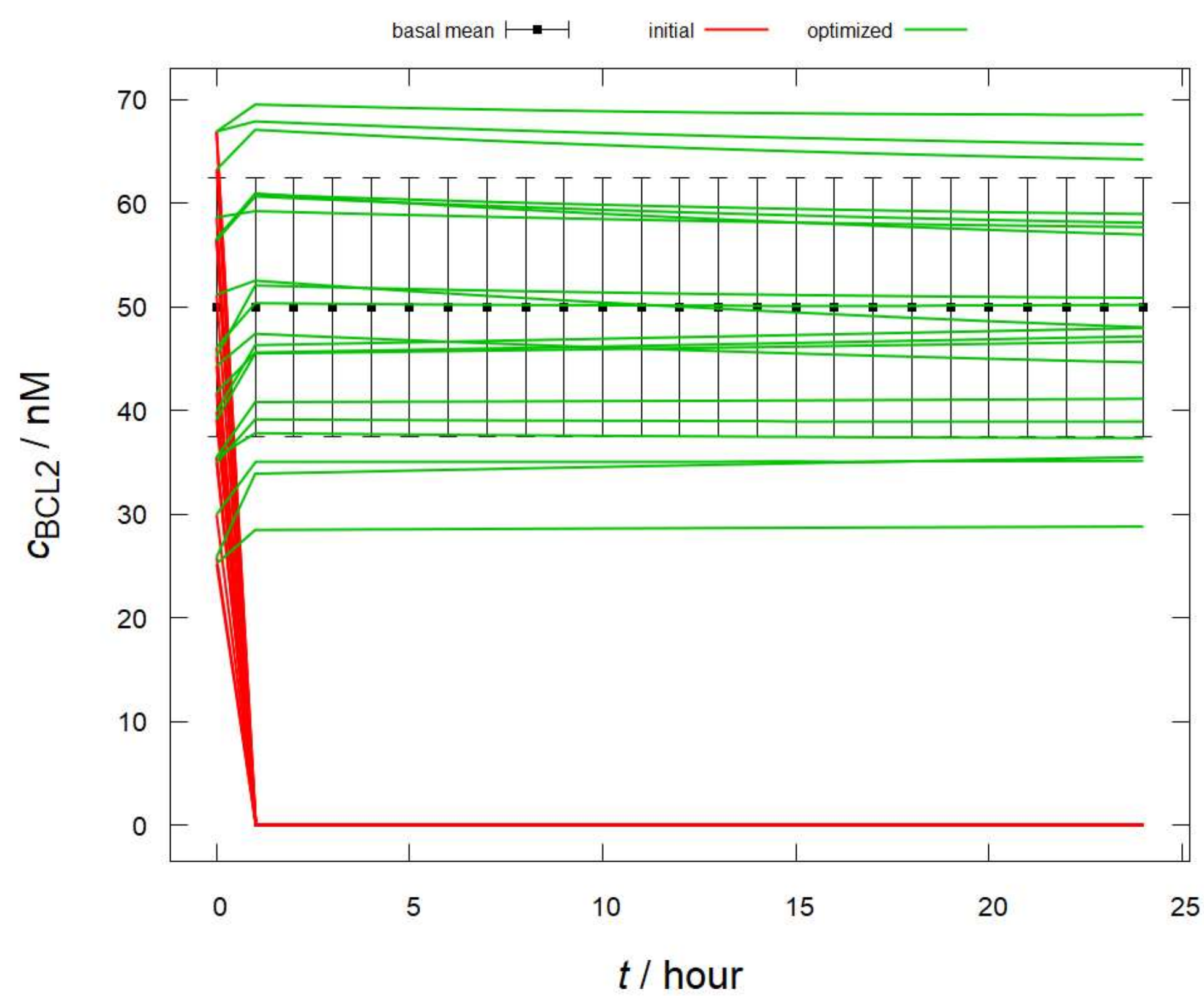

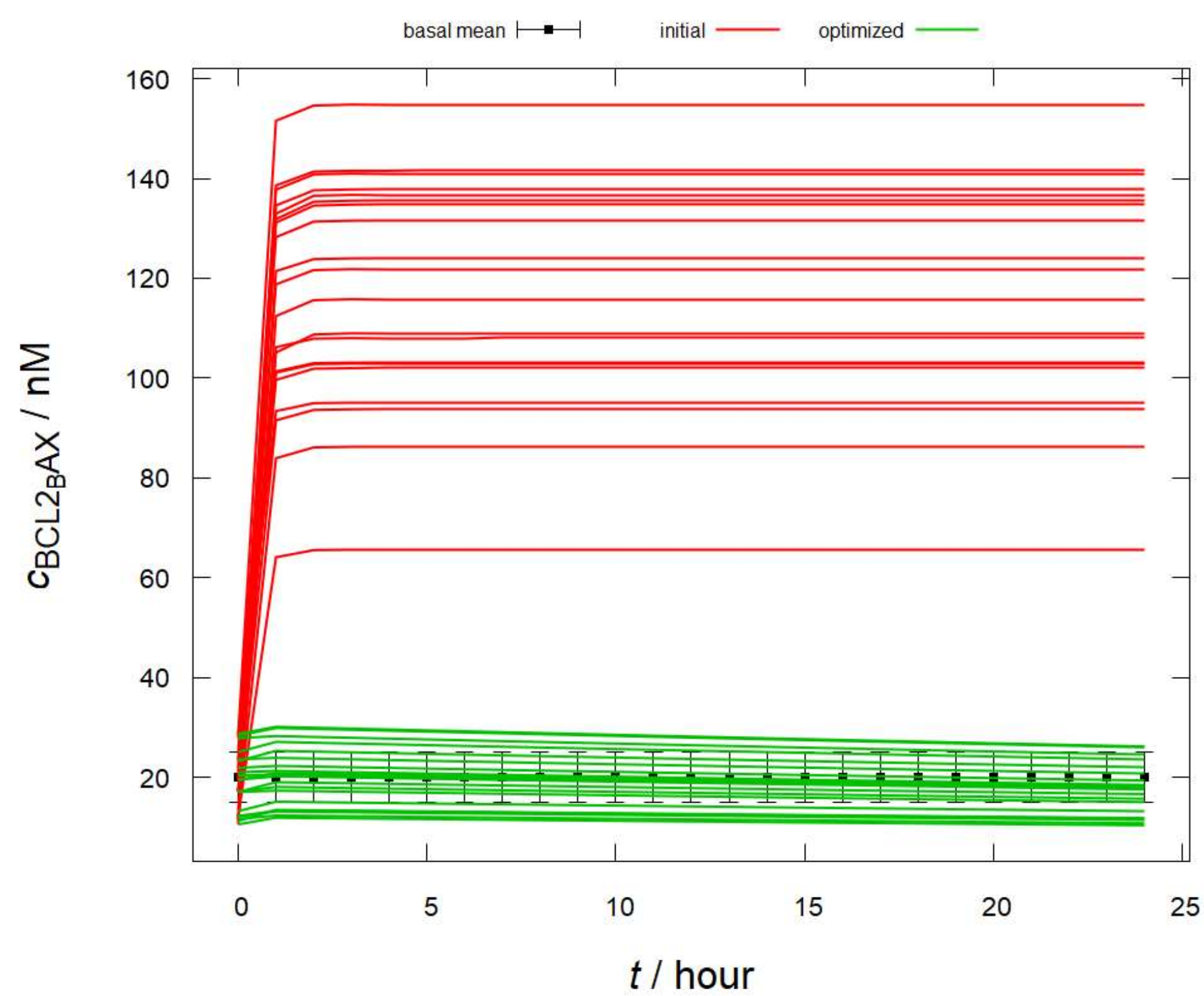

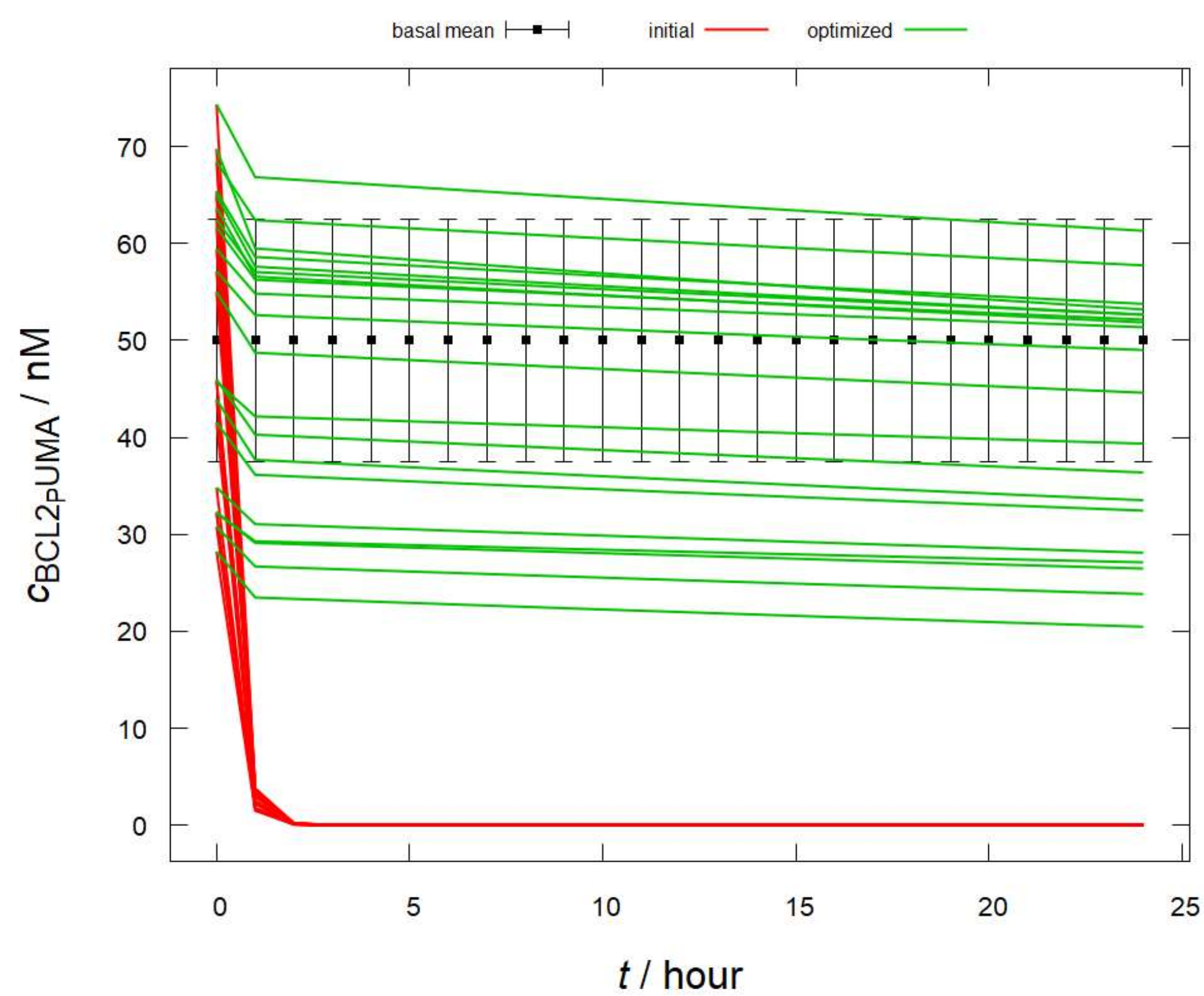

$c_{\text{BEC1}} / \text{nM}$

basal mean

initial

optimized

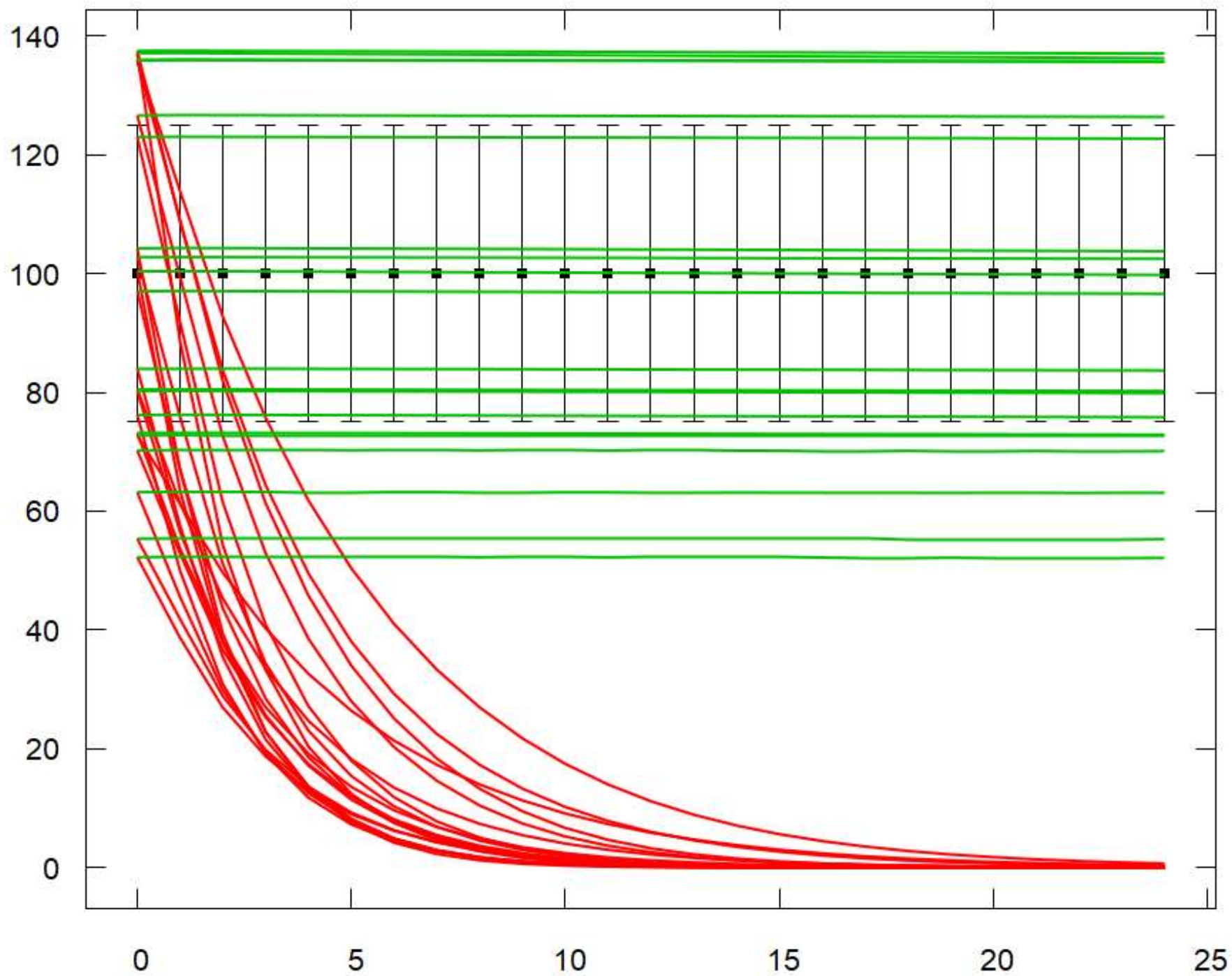

$t / \text{hour}$

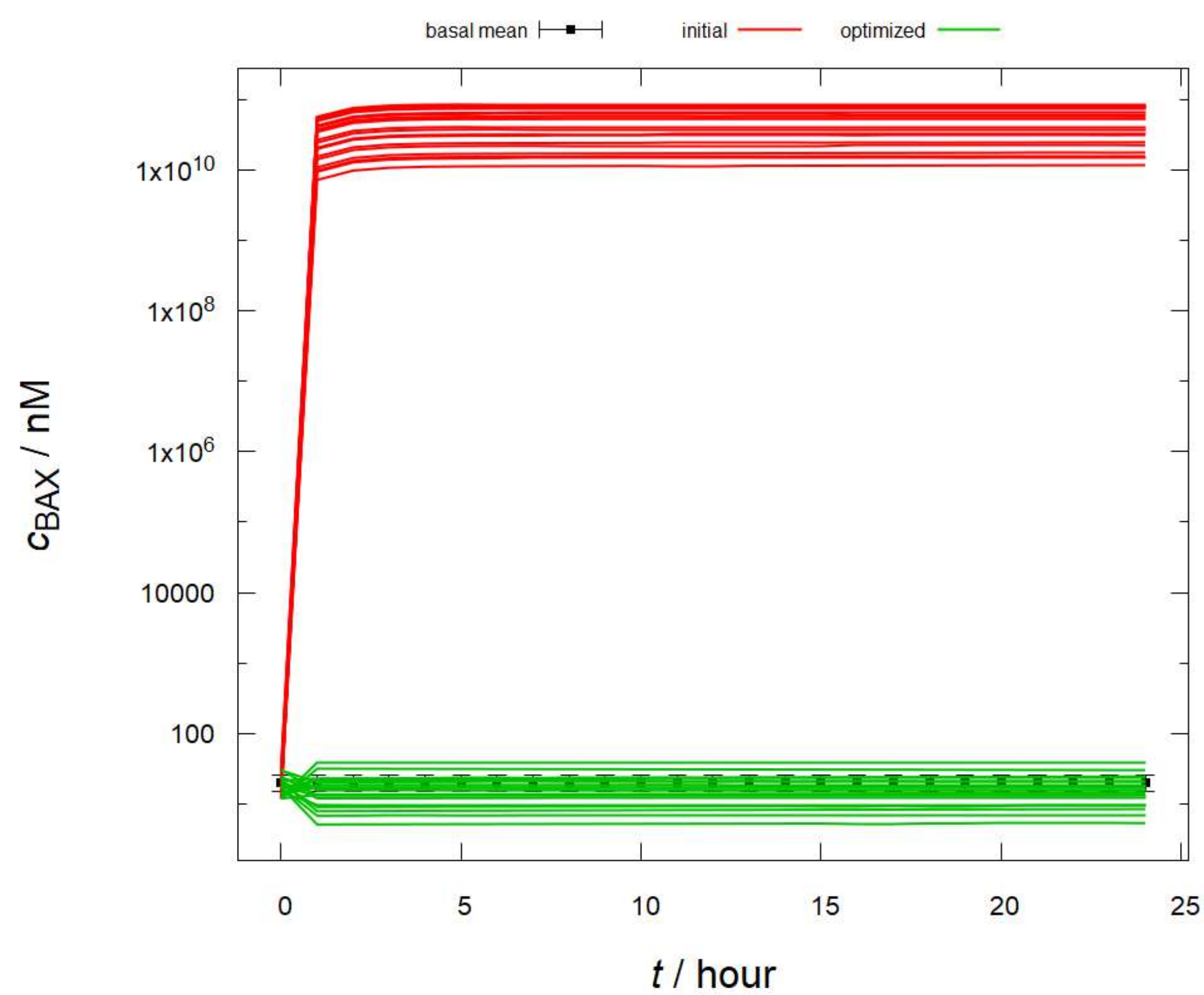

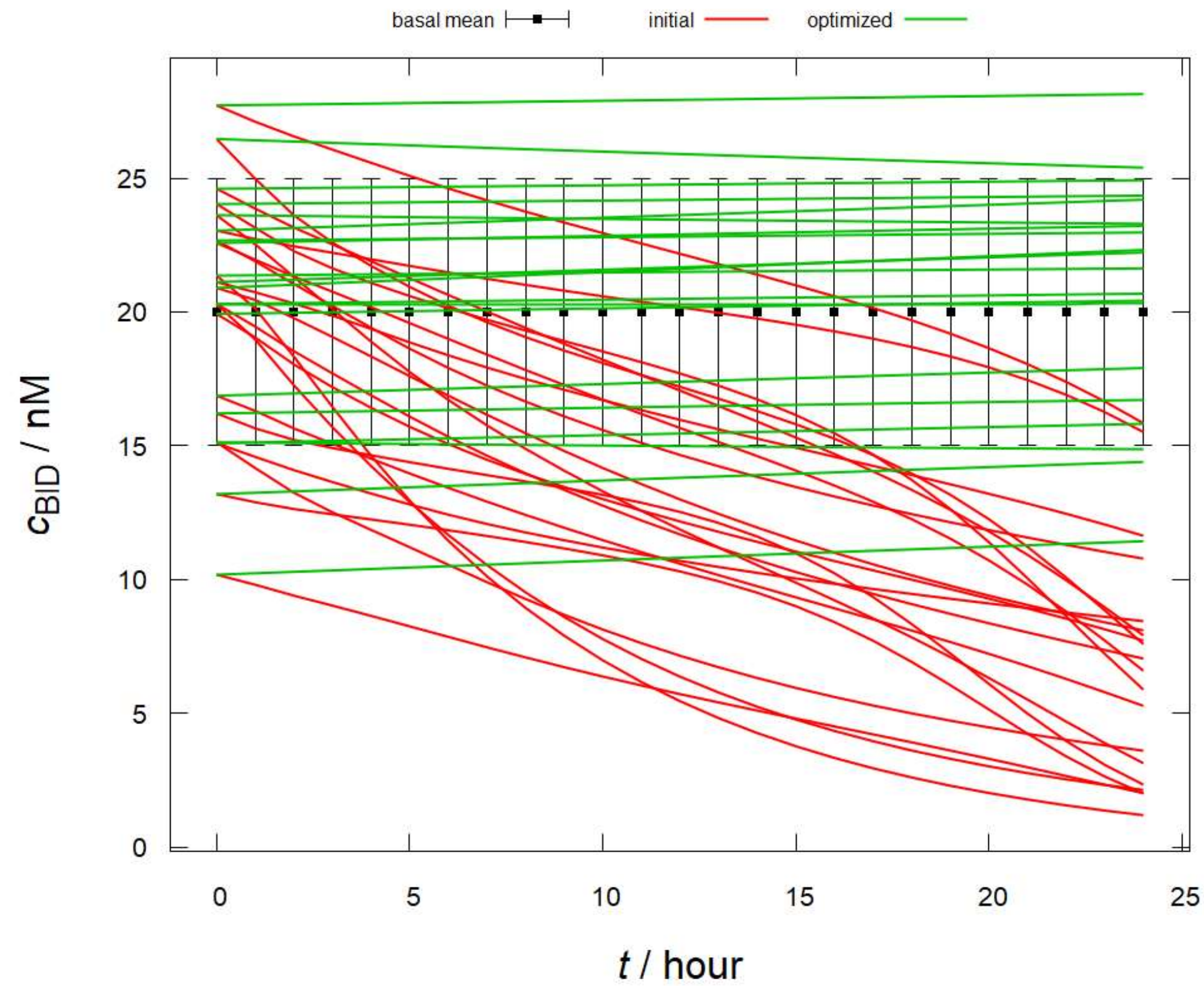

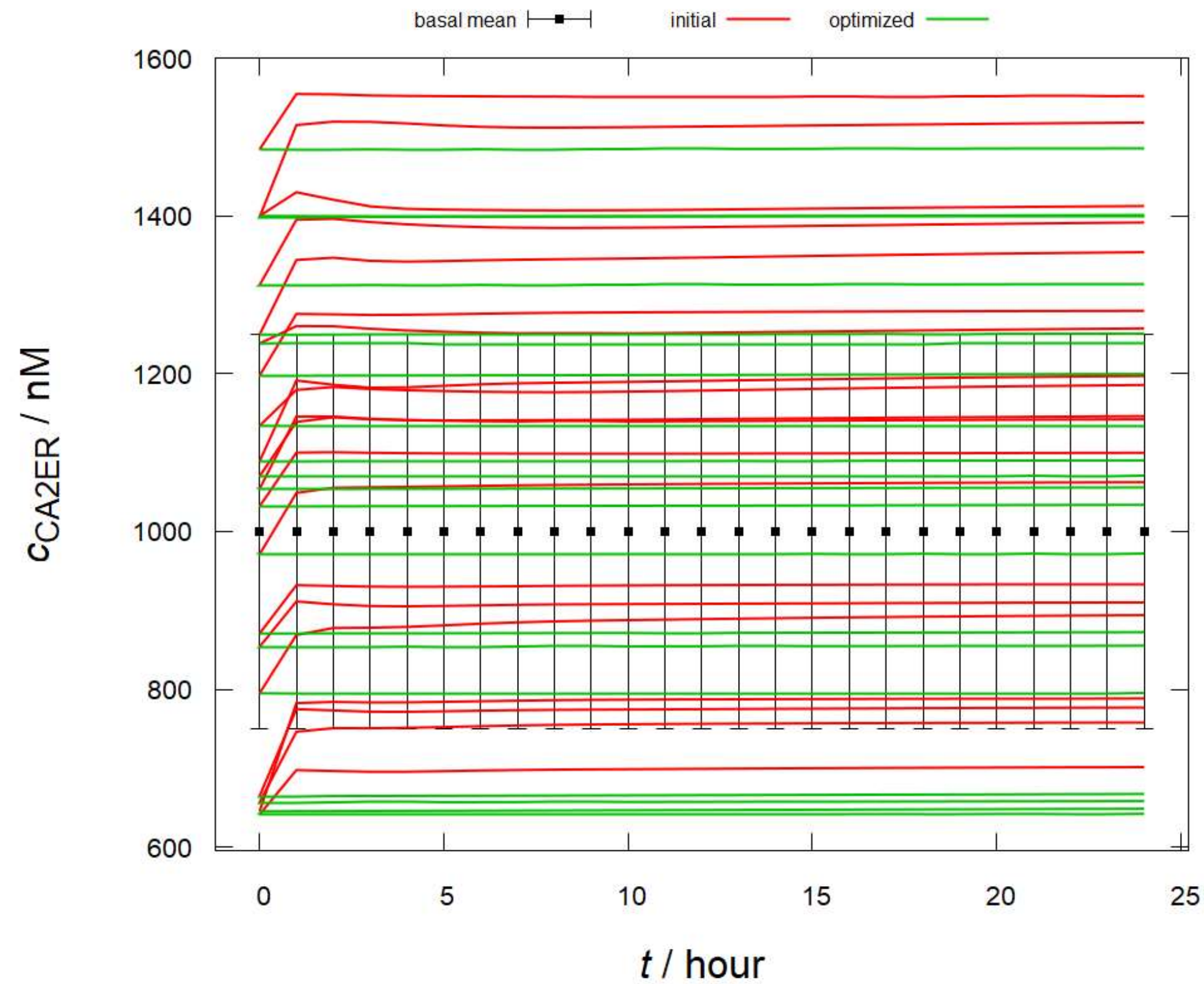

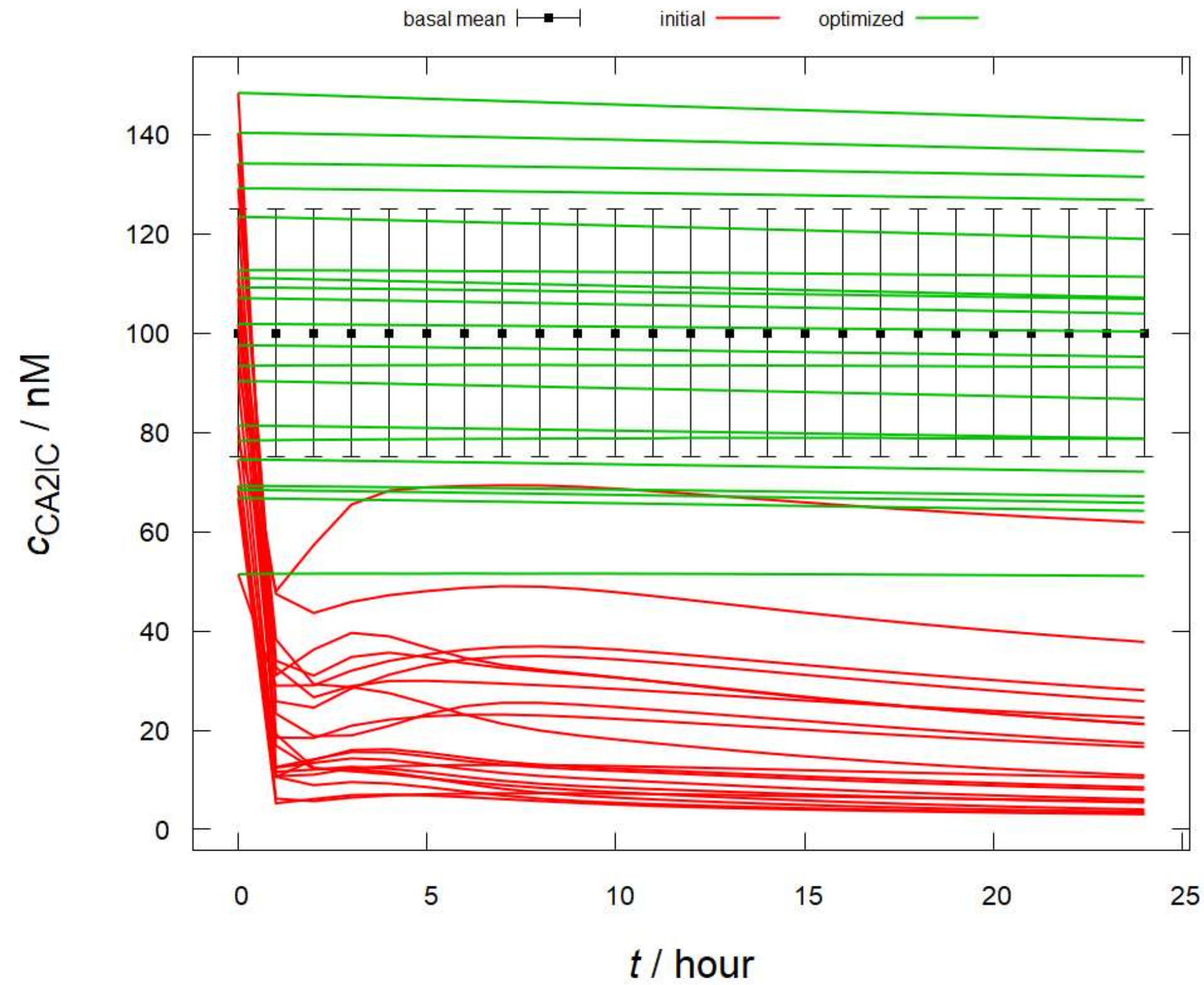

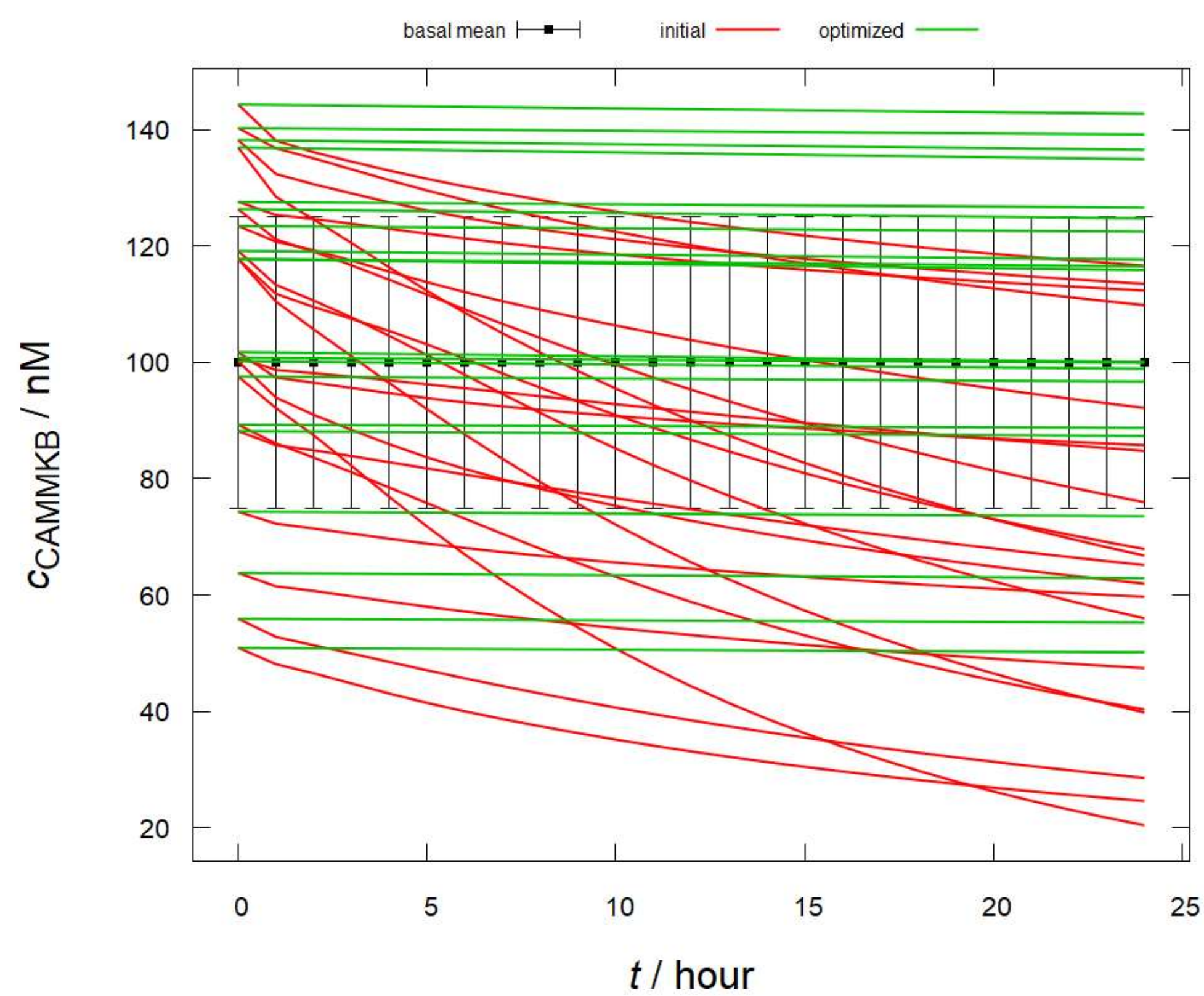

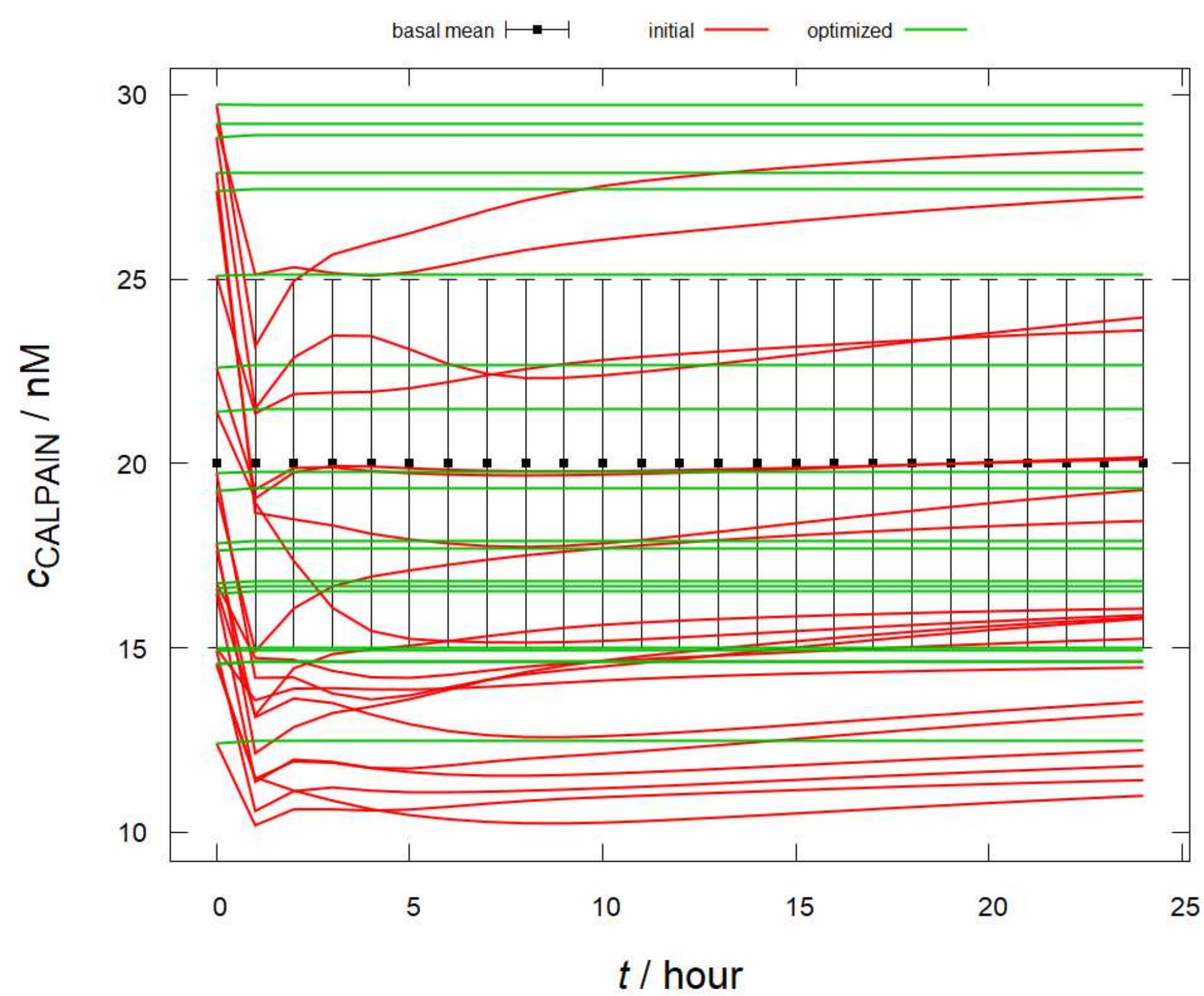

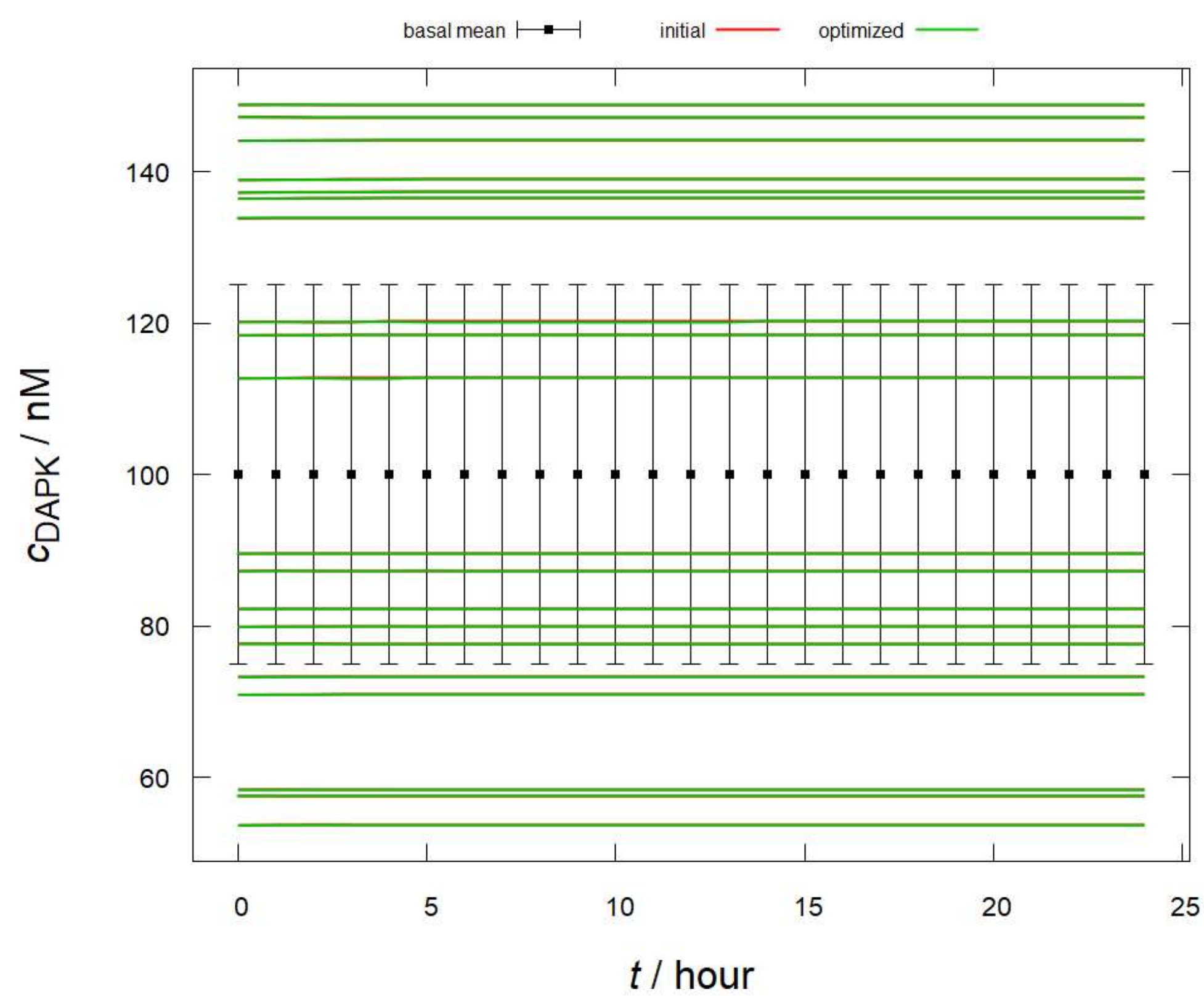

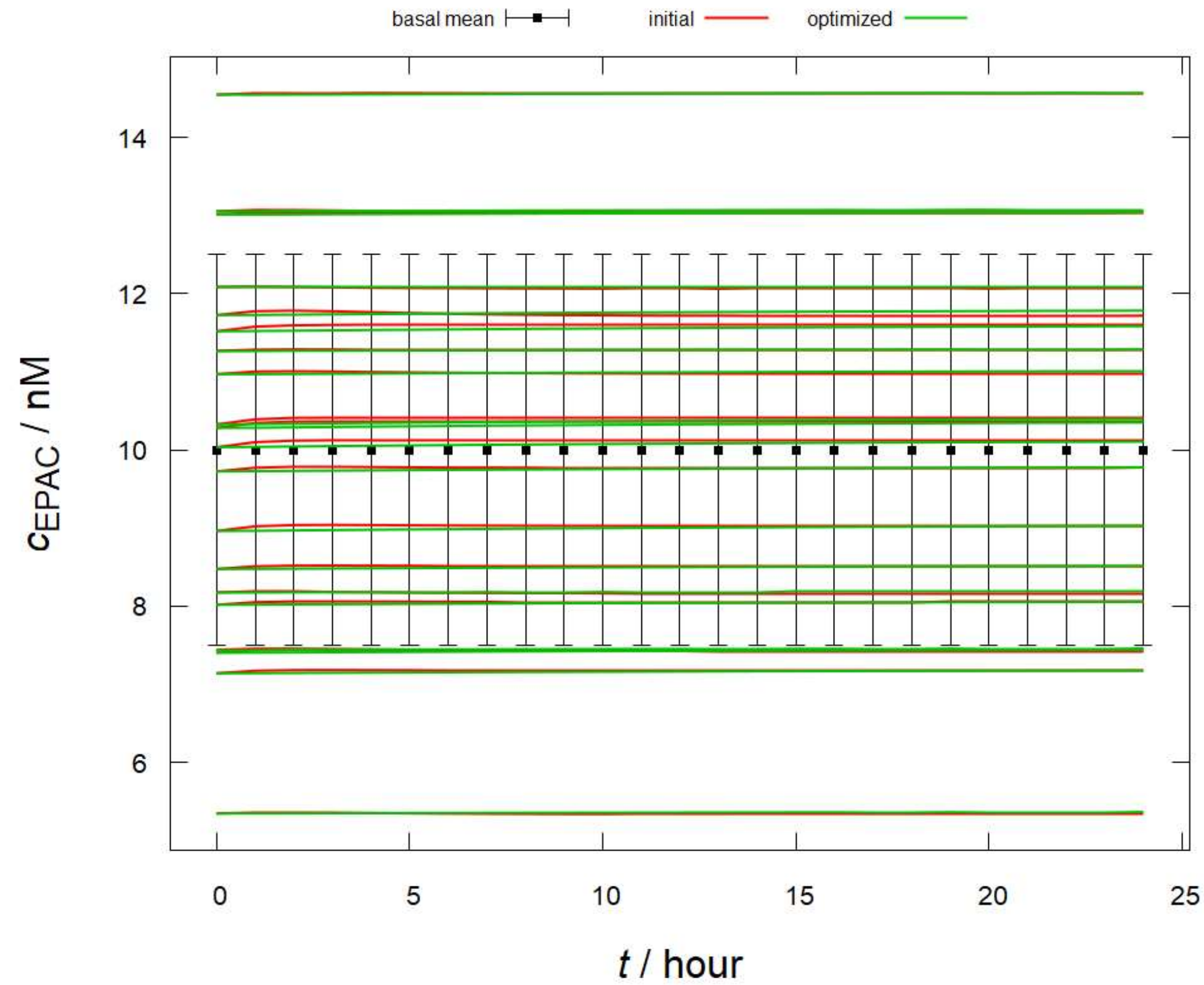

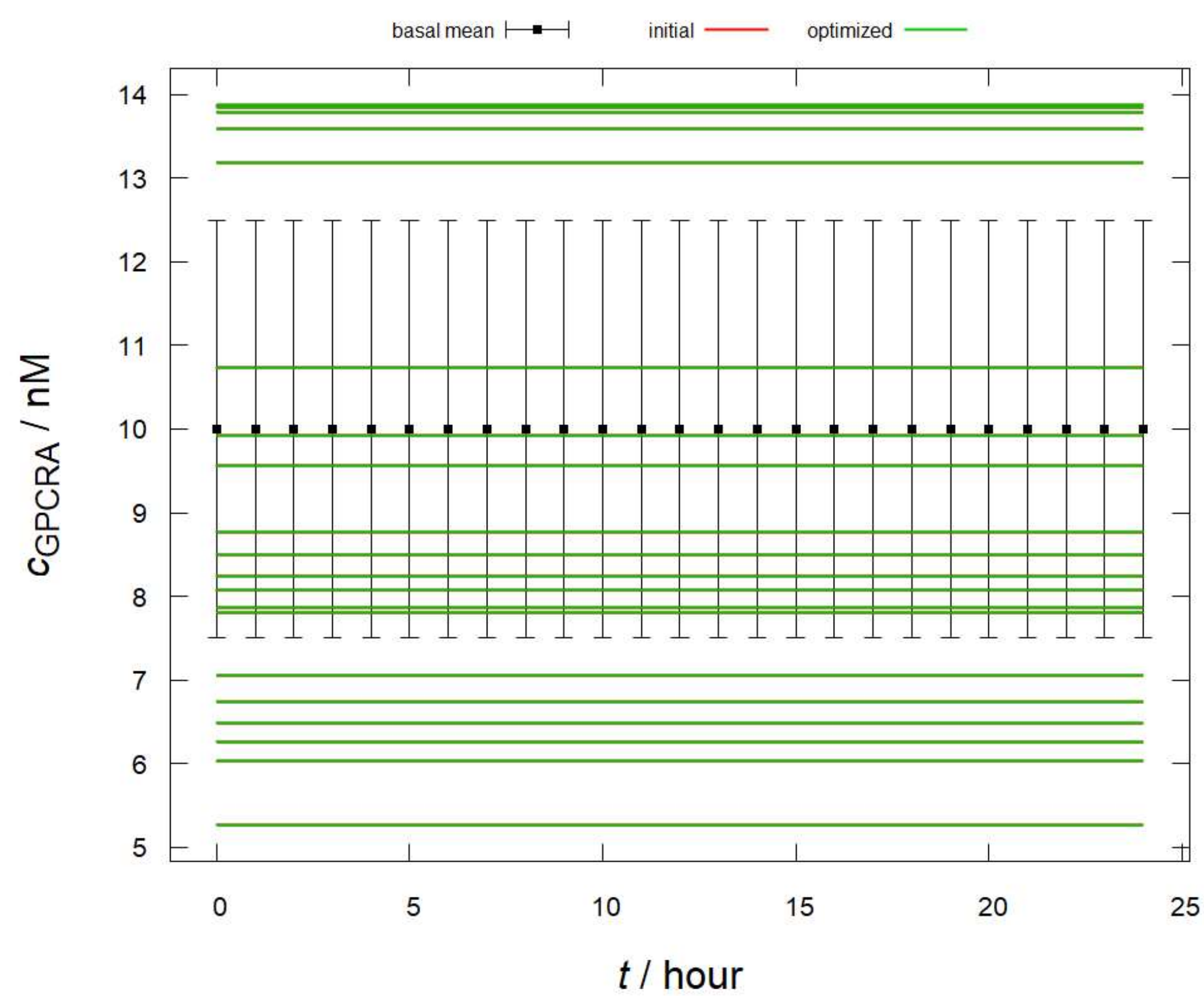

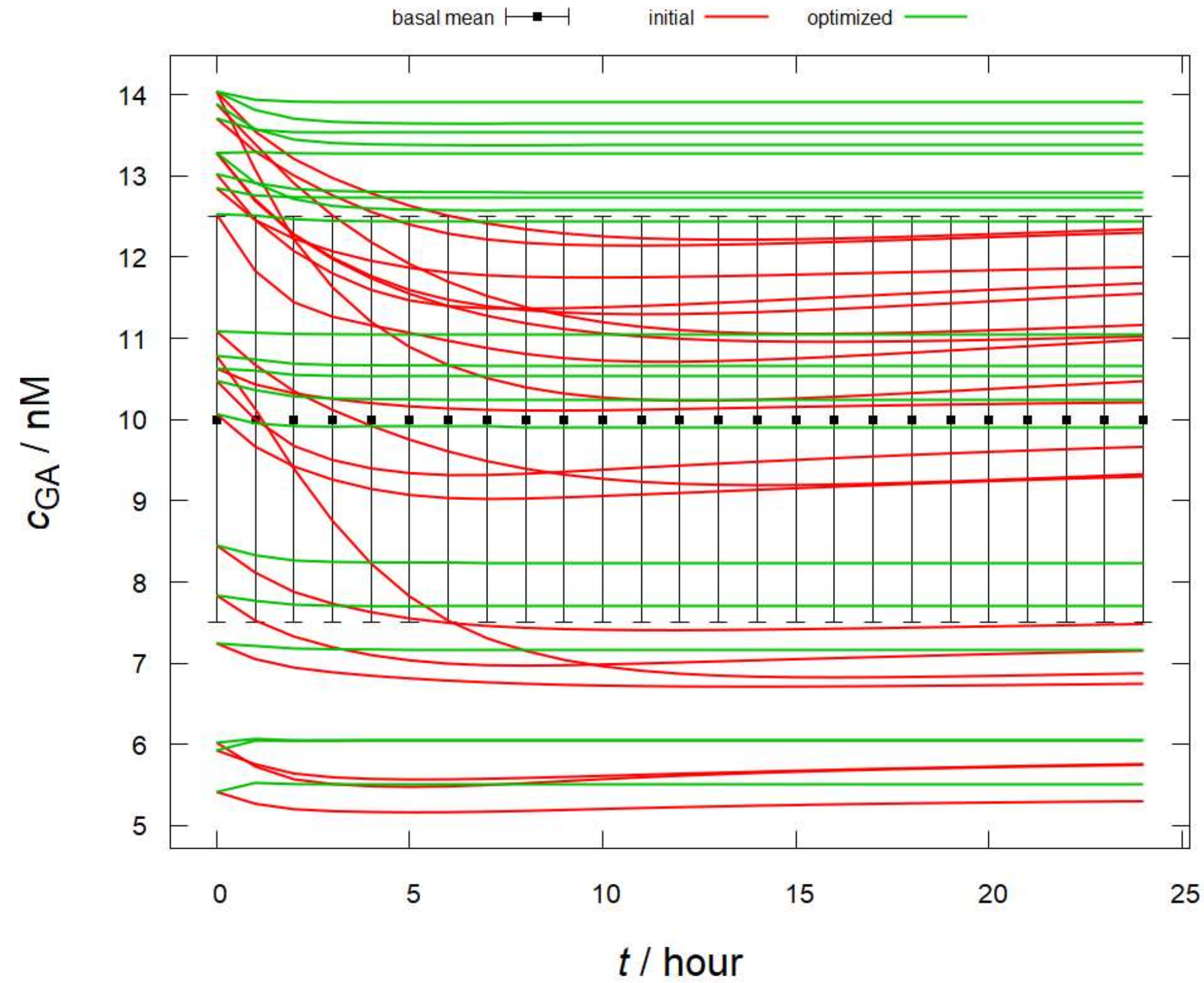

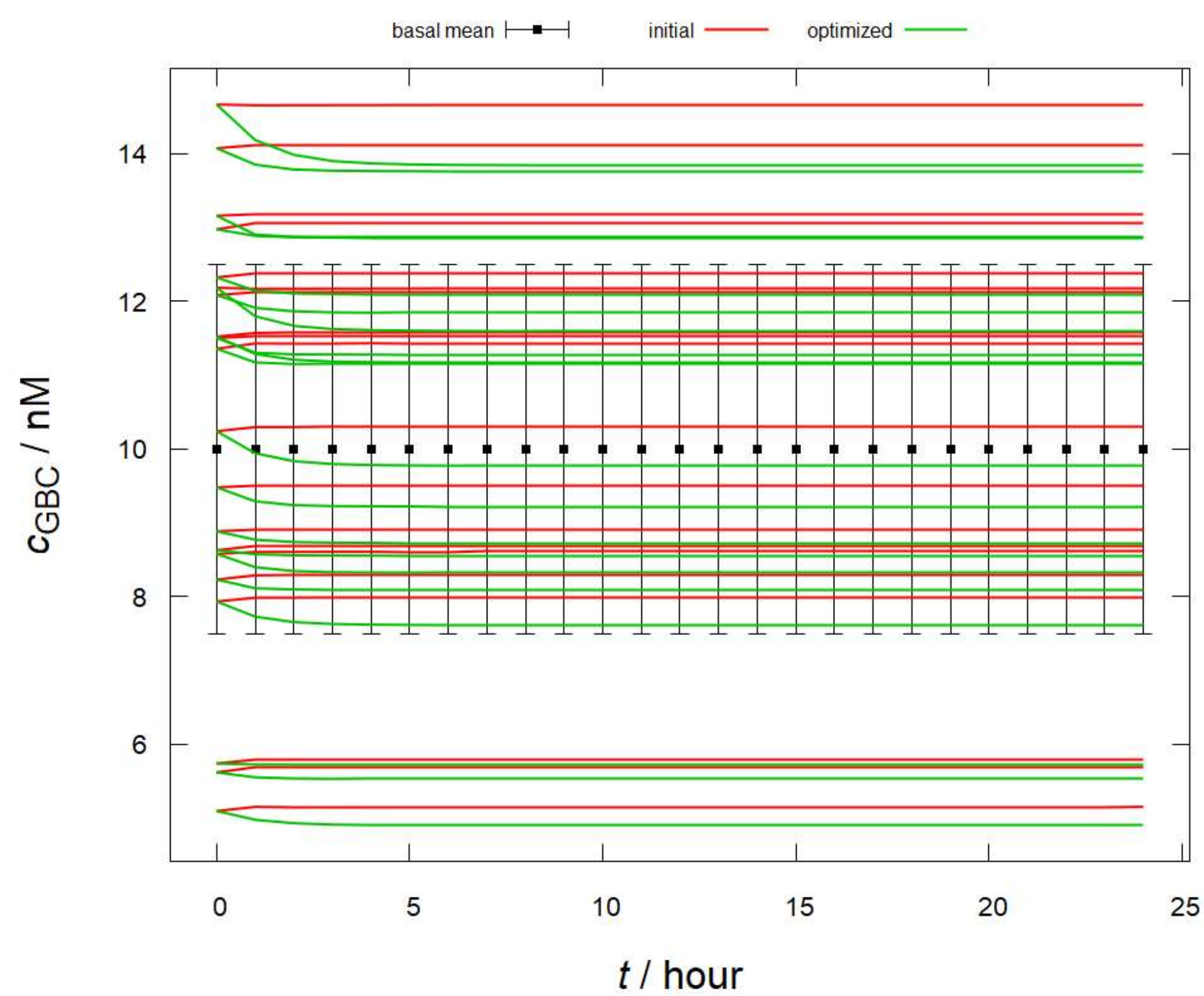

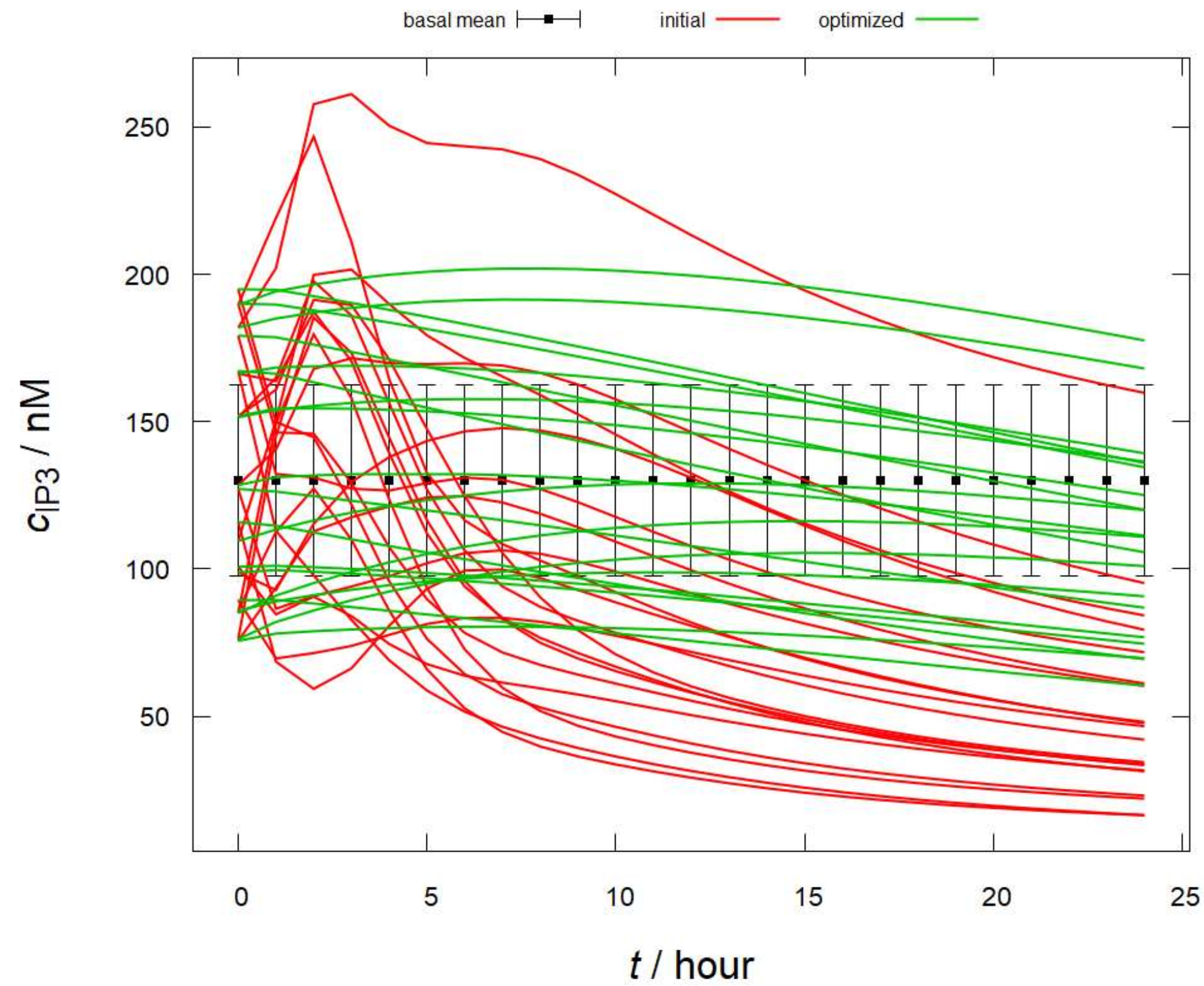

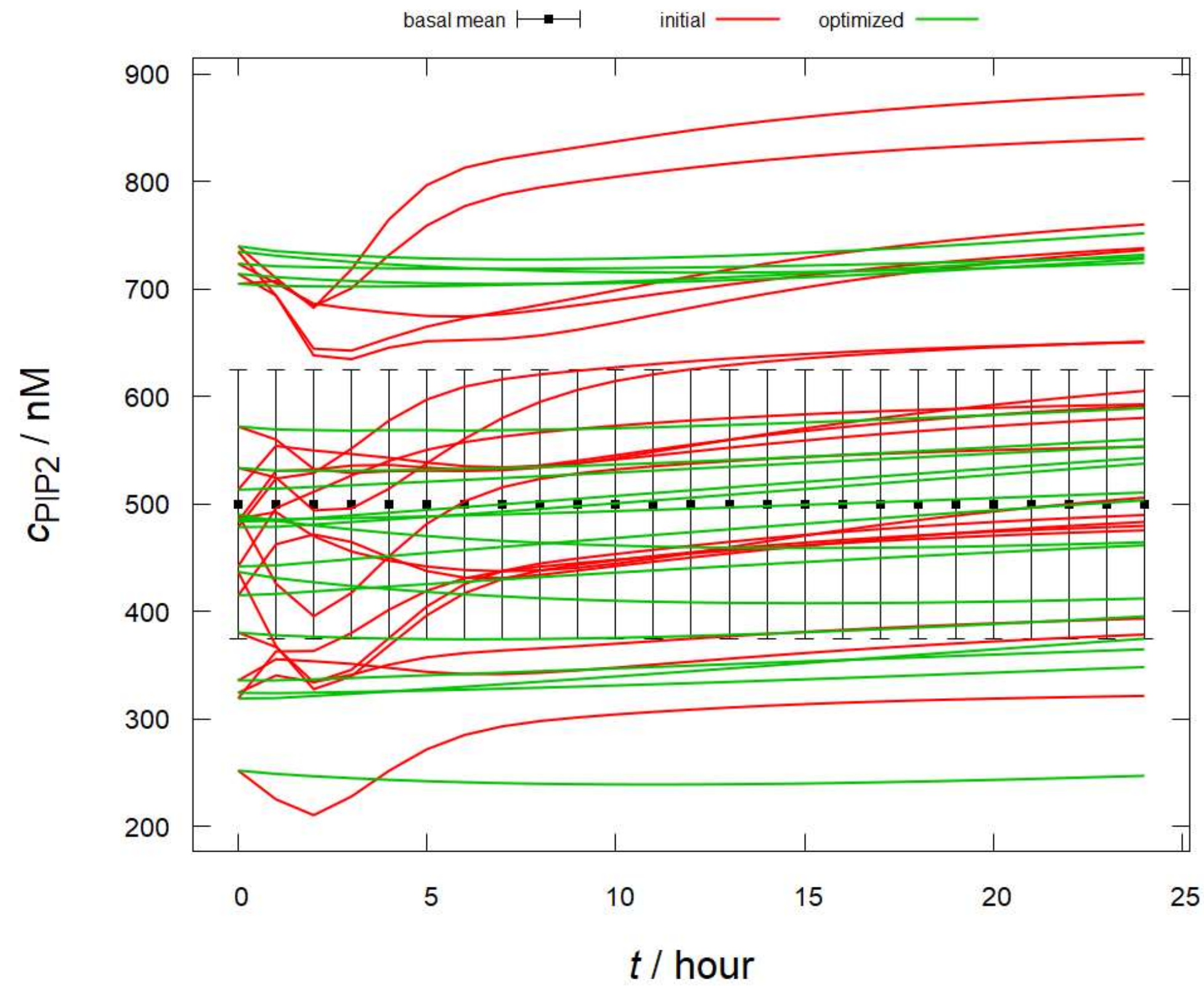

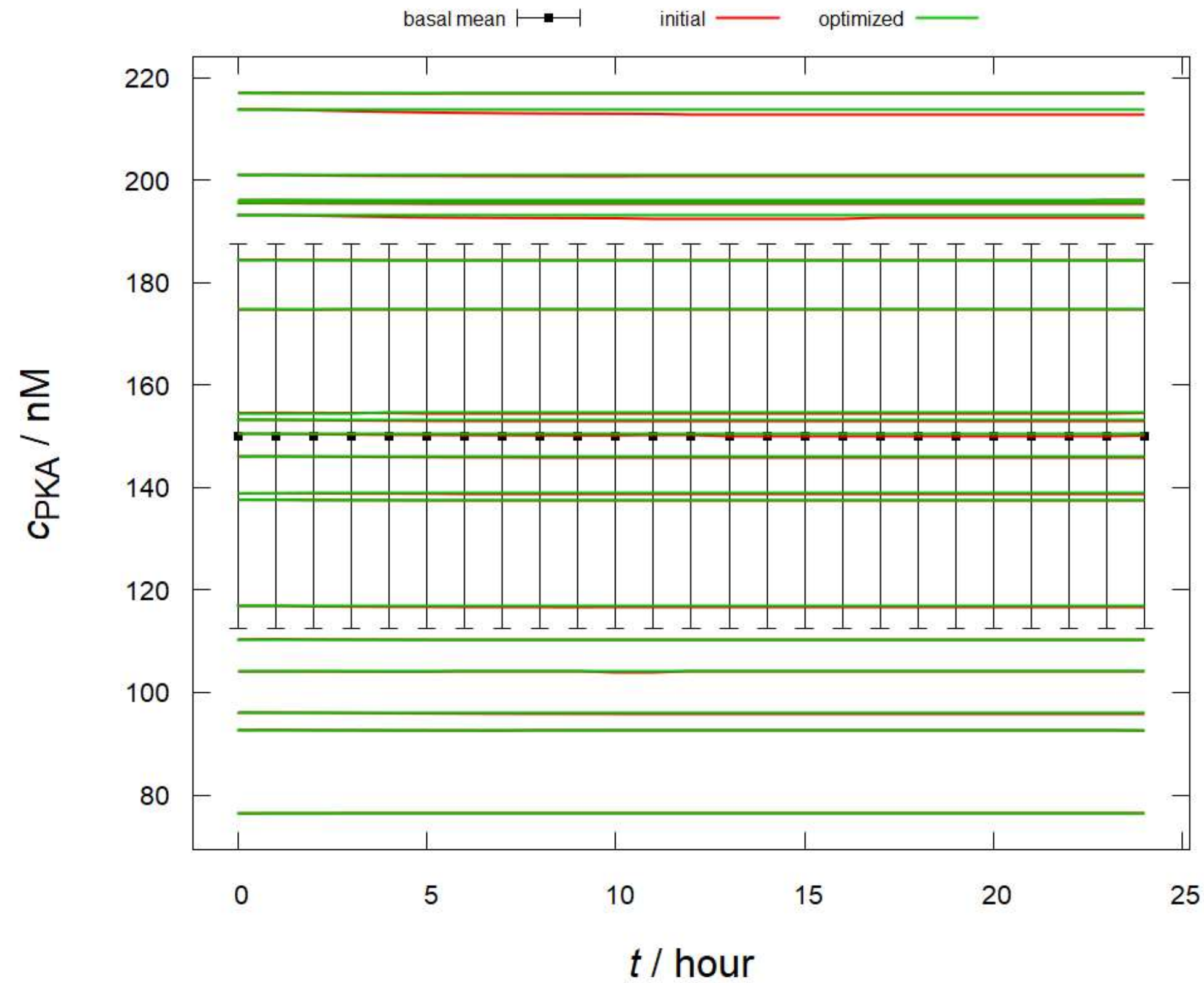

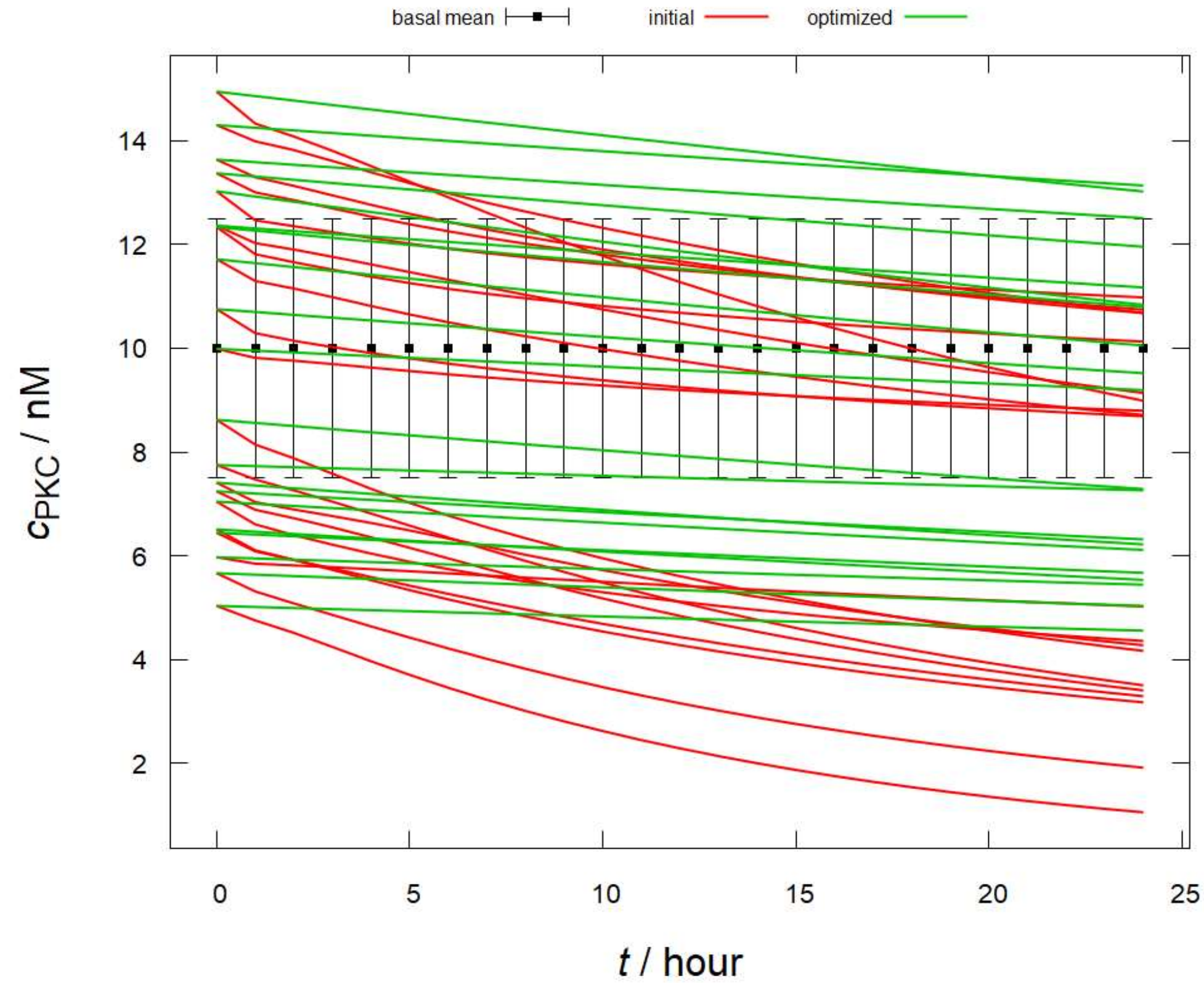

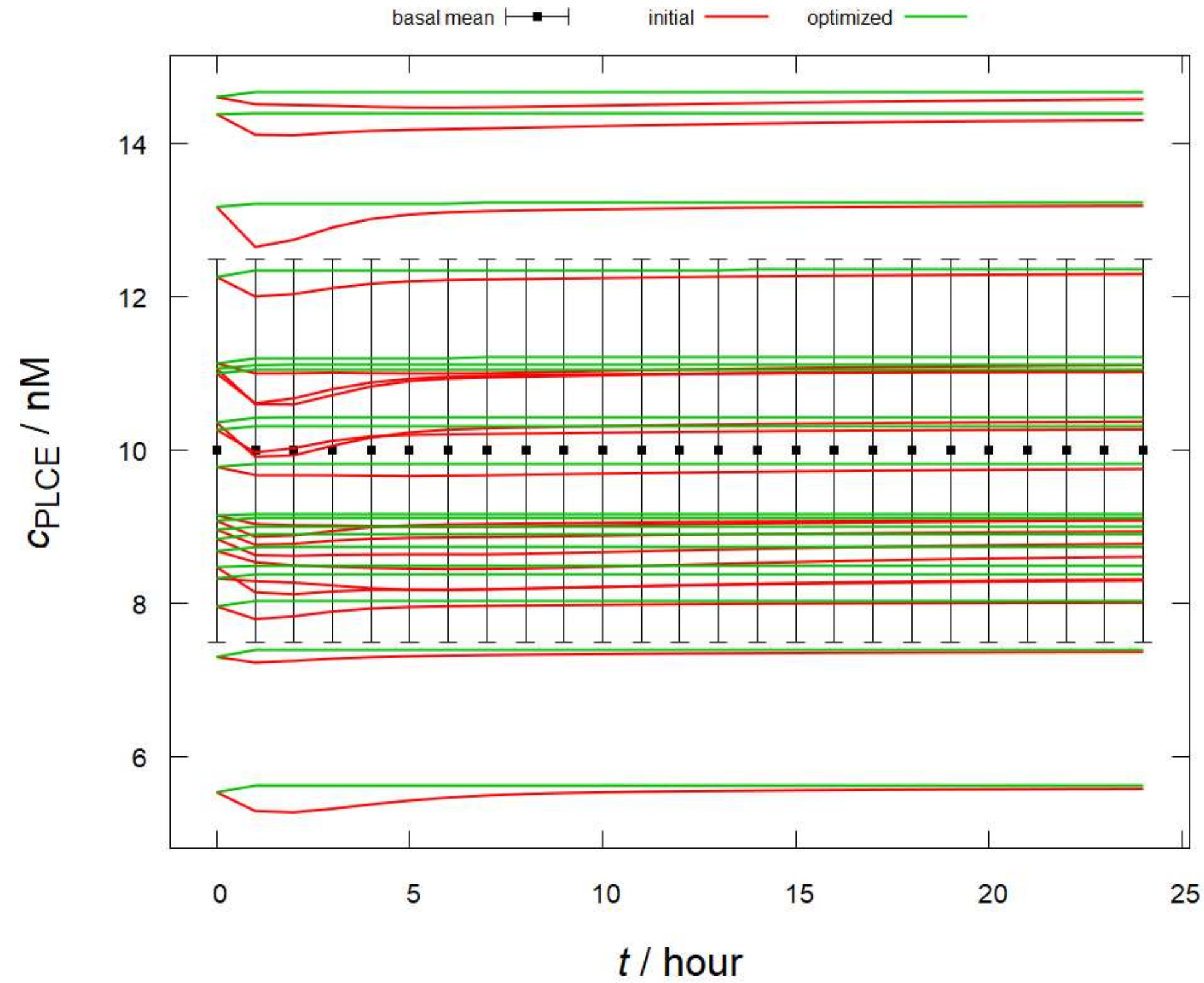

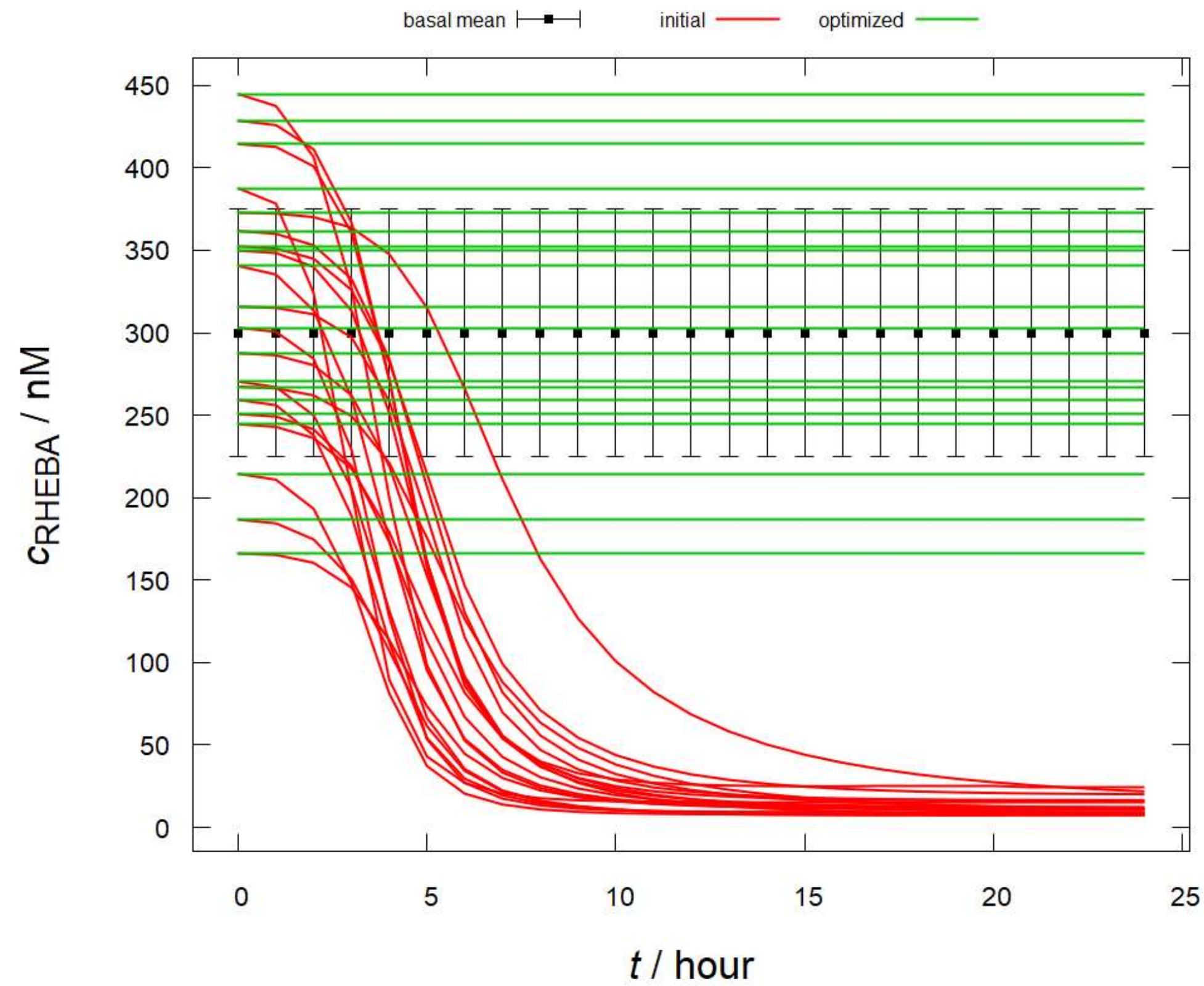

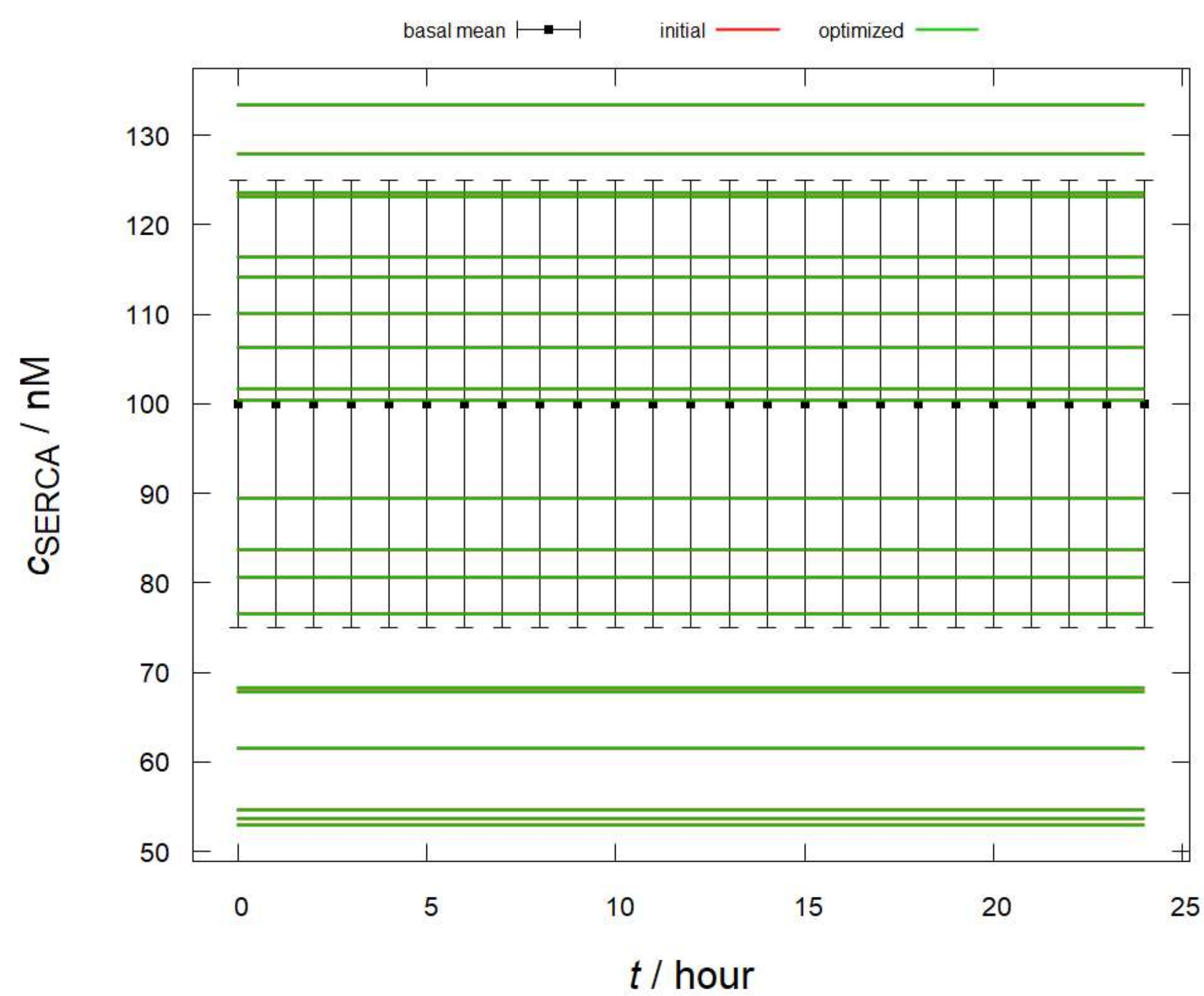

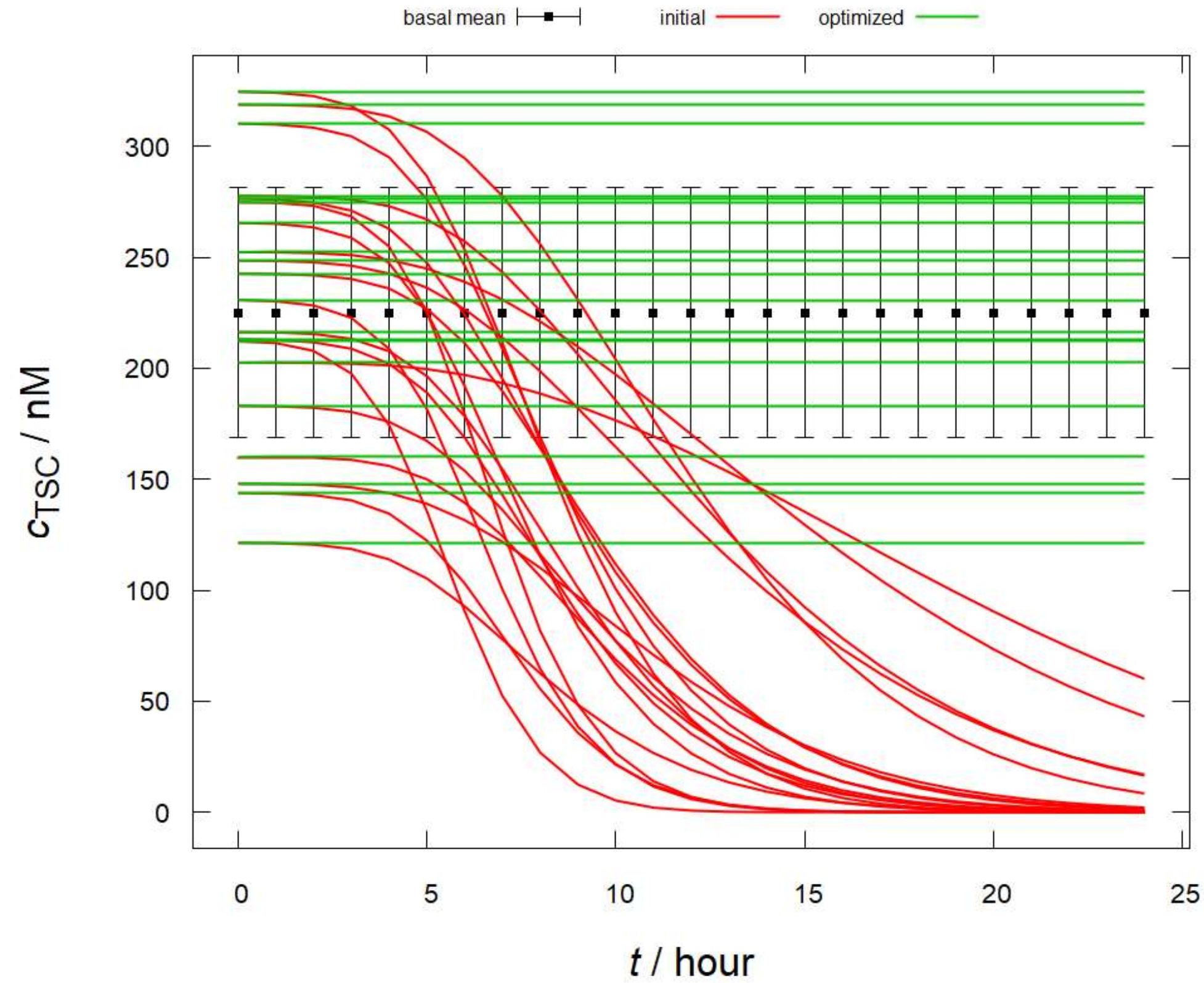

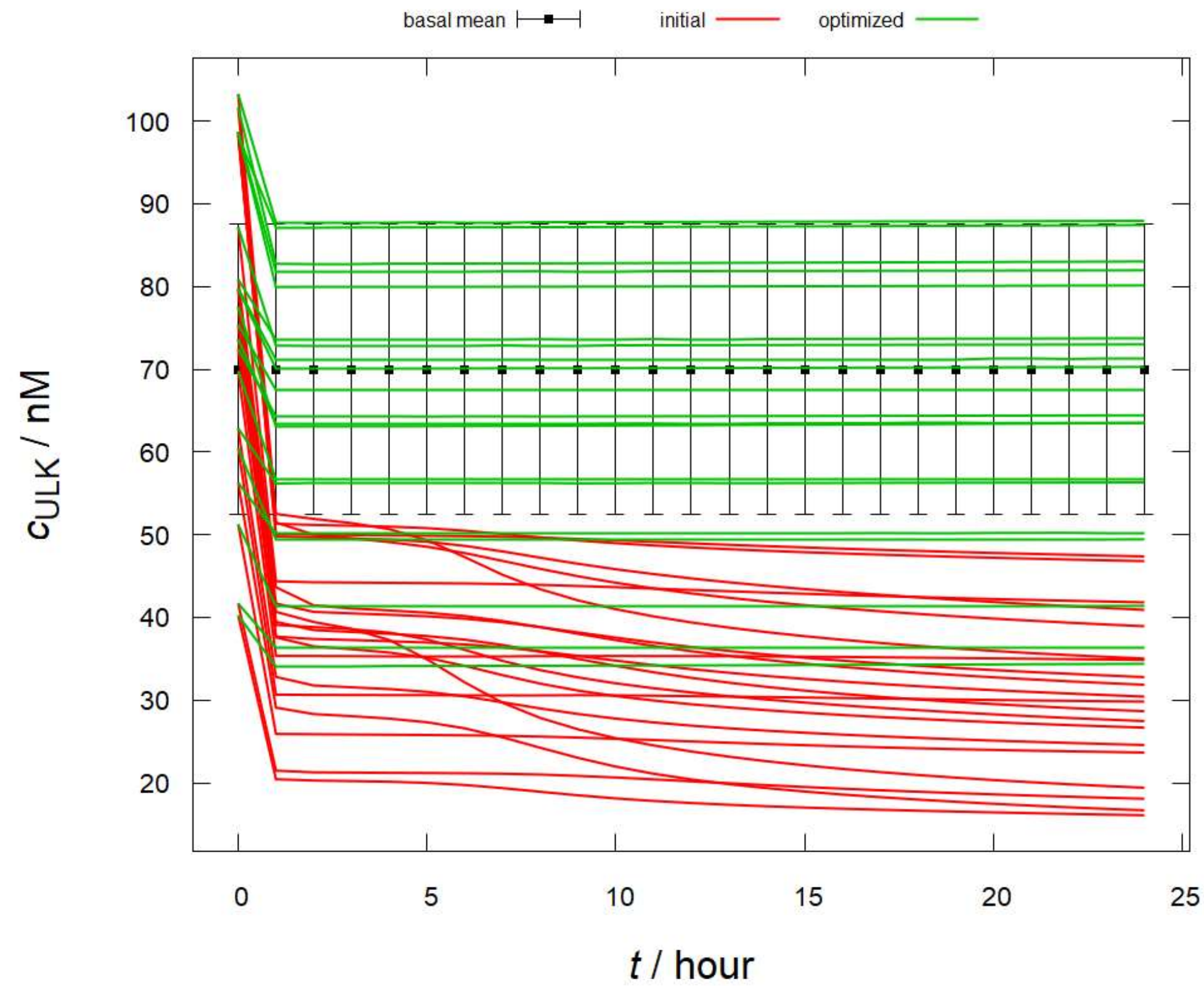

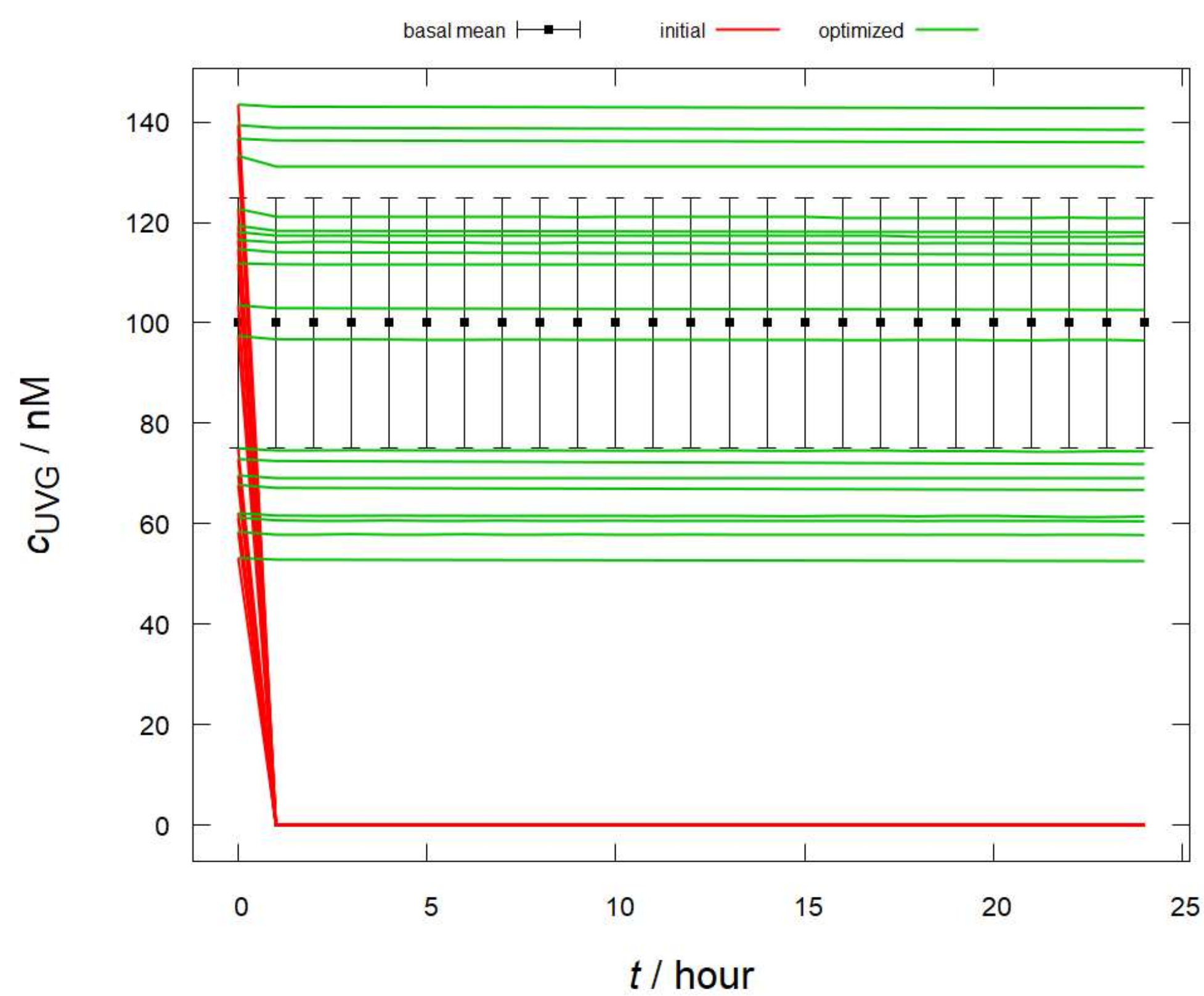

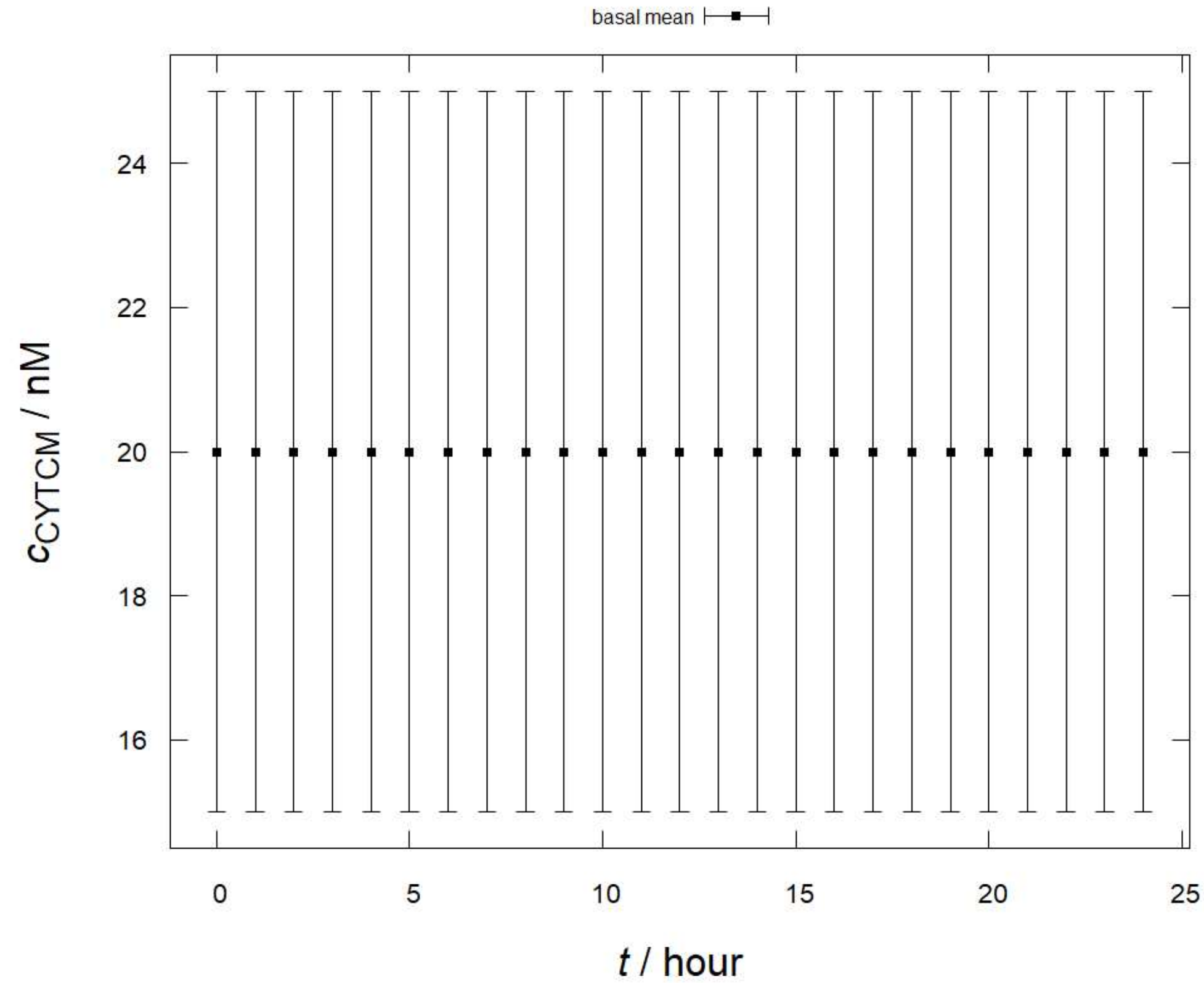

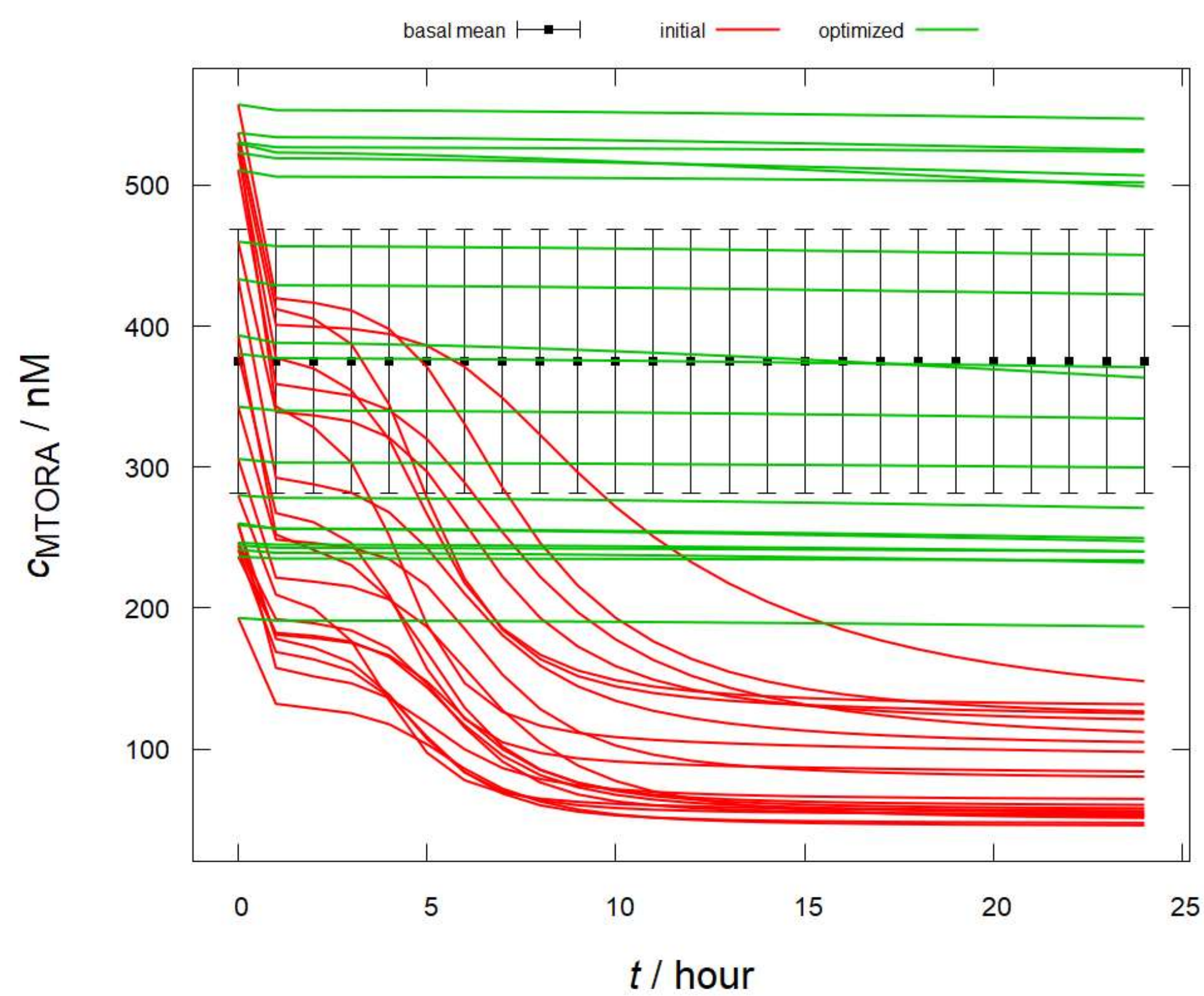

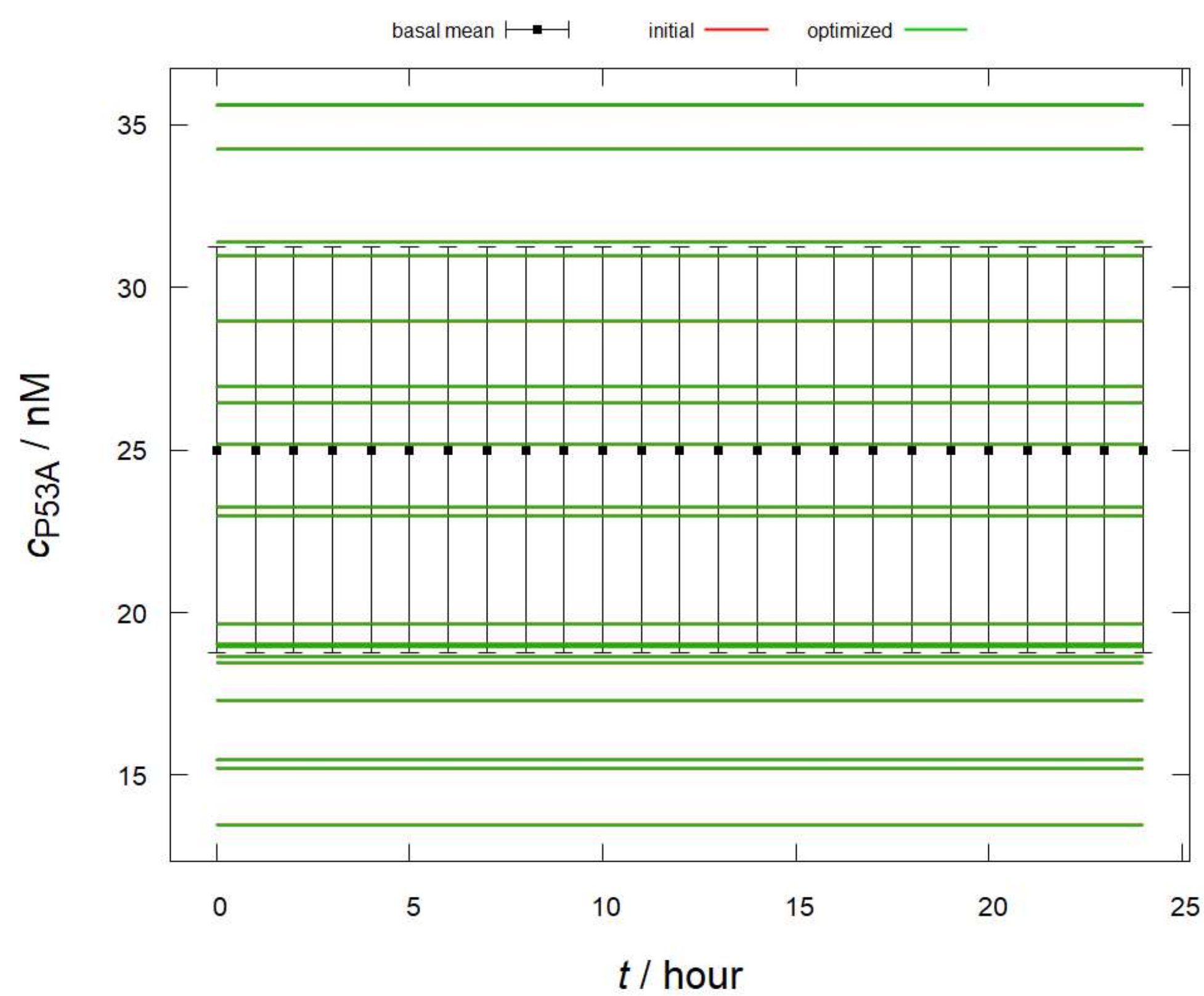

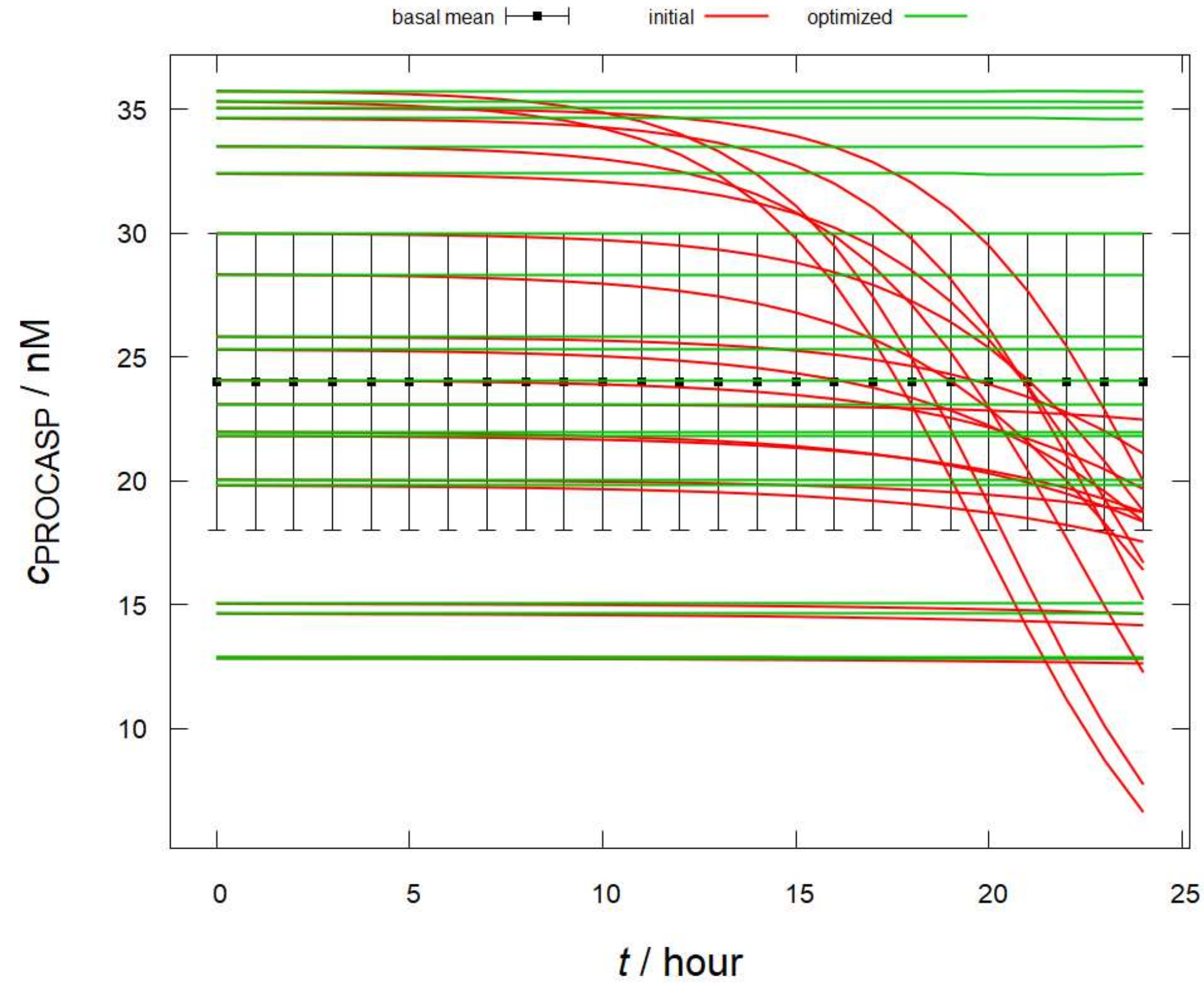

Supplement: Supplementary file 1 [file ijms-25-11316-s001.zip › supplement/all_species_plots.pdf]
